# ZOTMPo–INAR(1) Process: Entropy and Modeling of Epidemiological Count Time Series Data

**DOI:** 10.3390/e28080889

**Published:** 2026-08-07

**Authors:** Manik Awale, Shrirang Pund, Hassan S. Bakouch, Aishwarya Ghodake, Amira F. Daghestani, Souha K. Badr

**Affiliations:** 1 Department of Statistics, Savitribai Phule Pune University, Pune 411007, Maharashtra, India; manikawale@unipune.ac.in (M.A.); shrirang.p30@gmail.com (S.P.); ghodakeaish@gmail.com (A.G.); 2 Department of Mathematics, College of Science, Qassim University, Buraydah 51452, Saudi Arabia; 3 Department of Mathematics, College of Science and Humanities, Imam Abdulrahman Bin Faisal University, Jubail 35811, Saudi Arabia; adaghestani@iau.edu.sa; 4 Department of Mathematics and Statistics, College of Science, University of Jeddah, Jeddah 21959, Saudi Arabia; skbadr@uj.edu.sa

**Keywords:** count time series, zero–one–two inflation, autoregression, autocorrelation, forecasting, estimation, 62M10, 62P10

## Abstract

In this article, we propose a first-order integer-valued autoregressive (INAR(1)) model based on binomial thinning, in which the innovation sequence follows a zero–one–two-modified Poisson (ZOTMPo) distribution. The proposed model accommodates overdispersion and allows for inflation or deflation at low-count values, particularly at zero, one, and two, which are commonly observed in public health count time series. We derive the main probabilistic properties of the model and estimate the unknown parameters using the conditional maximum likelihood (CML) method. A Monte Carlo simulation study is conducted to evaluate the finite-sample performance of the estimators. Furthermore, we establish the information-theoretic properties of the model, specifically deriving the Shannon entropy and conditional entropy bounds to quantify the dynamical complexity and predictability of the stochastic process. The practical utility of the model is illustrated using two real-world datasets on dengue fever incidence and *Escherichia coli* (*E. coli*) enteritis. Model performance is assessed using standard information criteria and forecast accuracy measures, as well as the Euclidean distance between observed and fitted probabilities for zero, one, and two. Diagnostic checks, including analysis of residual autocorrelation, cumulative periodograms, and jump process behavior, provide further confirmation of the fitted model’s adequacy. The results indicate that the proposed ZOTMPo–INAR(1) model provides an effective framework for modeling overdispersed count time series with a modified low-count structure.

## 1. Introduction

Integer-valued time series models are widely used to analyze count data over time, particularly in epidemiology and public health surveillance, as well as in fields such as climatology and insurance. Such data are characterized by discreteness, serial dependence, overdispersion, and often inflation or deflation at specific low-count values (e.g., zero, one, and two), which are not adequately captured by standard Gaussian time series models. In the context of complex epidemiological systems, these integer-valued time series inherently represent discrete stochastic fluctuations and random noise driven by unpredictable environmental factors. Traditional models often struggle to capture the highly structured noise present in real-world dynamics, particularly the abrupt low-count variations and overdispersion. By framing the INAR process as a dynamic model of temporal fluctuations, the innovation sequence effectively captures the underlying stochastic noise that governs the system. Consequently, directly addressing inflation or deflation in specific smaller values, such as zero, one, and two counts, provides a refined mathematical approach to isolating true signals from complex systemic noise. This perspective not only enhances the accuracy of the forecasting of infectious disease dynamics but also directly aligns with the broader interdisciplinary pursuit of characterizing random fluctuations and noise in biological systems. To address these challenges, Refs. [1,2] introduced the integer-valued autoregressive (INAR) process. The INAR(1) model employs a thinning operator [3], typically binomial thinning, to capture dependence between successive observations while preserving the process’s integer nature. This framework has given rise to numerous extensions designed to capture more complex distributional characteristics of count data. Considerable research has focused on modifying the innovation distribution to address overdispersion and excess zeros. For example, INAR-type zero-modified and zero-inflated models have been widely studied, including those based on geometric, Poisson, and Poisson–Lindley distributions [4,5,6,7]. More recent contributions have extended these models to account for inflation at multiple values, such as zero and one, as well as bounded and multivariate count processes [8,9,10,11]. Additional developments include testing procedures for overdispersion and inflation, as well as models incorporating seasonal and periodic structures [12,13,14,15,16,17]. Despite significant progress in modeling zero- and one-inflated count time series, a critical gap remains in the literature on the nuanced stochastic behavior of real-world epidemiological data. Infectious disease dynamics, such as the spread of *E. coli* or dengue fever, frequently exhibit complex structural fluctuations that go beyond mere excess zeros and ones. In many practical scenarios, these systems exhibit simultaneous inflation or deflation across the first three count states (zero, one, and two), coupled with overarching overdispersion. Existing models, while effective for simpler inflation patterns, often lack the mathematical flexibility to accurately capture these multipoint low-count anomalies without compromising the distribution’s tail behavior.

This limitation highlights the need for a more refined autoregressive framework that can isolate true epidemiological signals from complex, low-count systemic noise. Addressing this multipoint modification is not merely a theoretical exercise; it is crucial for accurate forecasting and reliable decision-making in public health. Therefore, developing a model that specifically accommodates simultaneous variations at zero, one, and two counts while preserving a standard Poisson structure for higher values provides a highly realistic and mathematically rigorous solution to the analytical shortcomings of current INAR methodologies.

To address this critical gap, in this article, we propose a novel INAR(1) model in which the innovation sequence follows a zero–one–two-modified Poisson (ZOTMPo) distribution. The proposed innovation distribution captures inflation or deflation at the three smallest count values while retaining a Poisson-type tail behavior.

The main contributions of this paper are as follows. First, we develop the ZOTMPo–INAR(1) model and derive its key probabilistic properties. Second, we estimate the model parameters using the conditional maximum likelihood (CML) method. Third, we conduct a Monte Carlo simulation study to evaluate the finite sample performance of the estimators. Finally, we demonstrate the practical utility of the proposed model using two real datasets on *E. coli* enteritis and dengue fever, and compare its performance with existing INAR-type models. The results indicate that the proposed model provides an improved fit, particularly in the presence of low-count inflation. Furthermore, to rigorously quantify the uncertainty and predictability of this complex system, we incorporate an information–theoretic approach by evaluating the Shannon entropy and the conditional entropy of the proposed process. This entropy-based perspective provides a deeper understanding of the structural complexity driven by multipoint low-count modifications.

The proposed model is intended for count time series in which the empirical frequencies of counts 0, 1, and 2 differ noticeably from those expected under a standard Poisson distribution. Such departures may arise because of inflation or deflation at these low-count values. In these situations, the proposed innovation distribution provides an alternative to the classical Poisson innovation. The model is not intended to replace the Poisson INAR(1) model in general; rather, it is designed for datasets exhibiting these specific distributional characteristics. While the proposed ZOTMPo–INAR(1) model focuses on discrete-time count processes, the mathematical challenge of capturing complex, state-dependent fluctuations and structural noise is a pervasive theme across broader stochastic modeling. For instance, continuous-time heterogeneous diffusion processes with spatial or temporal disorder show that changes in the diffusion mechanism can affect the behavior of the process, leading to transient trapping, anomalous diffusion, and weak ergodicity breaking [18]. Similarly, in the discrete epidemiological models considered here, the innovation sequence represents the source of randomness. By allowing inflation or deflation at the low-count states, the ZOTMPo innovation modifies the stochastic behavior of the INAR(1) process. Although the two models arise in different settings, both illustrate how changes in the underlying random mechanism can influence the overall behavior of the stochastic process.

The remainder of the paper is organized as follows. Section 2 introduces the zero–one–two-modified Poisson (ZOTMPo) distribution, including its formulation and key distributional properties. Section 3 presents the proposed INAR(1) model with ZOTMPo innovations and discusses its stochastic structure and theoretical properties. Information–theoretic properties of the process are discussed in Section 4. Section 5 describes the parameter estimation procedure based on conditional maximum likelihood and reports the results of the simulation study. Section 6 illustrates the application of the proposed model to real-world datasets and provides a comparative performance analysis with existing count time series models. Finally, Section 7 concludes the paper.

## 2. Zero–One–Two-Modified Poisson Innovations and INAR(1) Model

### Zero–One–Two-Modified Poisson (ZOTMPo) Distribution

The ZOTMPo distribution is a mixture distribution that modifies the probabilities at lower counts (zero, one, and two). Let ε denote a discrete random variable following a ZOTMPo distribution, denoted as ε∼ZOTMPo(ϕ,λ,p), with parameters ϕ∈(0,1), λ>0, and p∈(0,1). It is defined as follows:

(1) $$\varepsilon =\left\{\begin{array}{cc} Bin(2,p), & \mathrm{with}\;\mathrm{probability}\;\phi ,\\ \mathrm{Pois}(\lambda ), & \mathrm{with}\;\mathrm{probability}\;1-\phi . \end{array}\right.$$

The corresponding probability mass function (PMF) of ε is given by

(2) $$P\left(\varepsilon =i\right)=\left\{\begin{array}{cc} \phi {\left(1-p\right)}^{2}+\left(1-\phi \right)e^{-\lambda }, & \mathrm{if}\;i=0,\\ 2\phi p\left(1-p\right)+\left(1-\phi \right)\lambda e^{-\lambda }, & \mathrm{if}\;i=1,\\ \phi p^{2}+\left(1-\phi \right)\frac{\lambda^{2}e^{-\lambda }}{2}, & \mathrm{if}\;i=2,\\ \left(1-\phi \right)\frac{\lambda^{i}e^{-\lambda }}{i!}, & \mathrm{if}\;i\geq 3. \end{array}\right.$$

This distribution modifies the probabilities at counts 0, 1, and 2, while retaining the Poisson form for counts i≥3. Depending on the parameter values, it can accommodate either inflation or deflation at the lower counts.

When ϕ=0, the ZOTMPo distribution reduces to the Poisson distribution with parameter λ. As ϕ increases, probability mass is redistributed among the counts 0, 1, and 2, while the probabilities for counts greater than 2 retain the Poisson form scaled by (1−ϕ). Consequently, the distribution departs from the moment characteristics of the Poisson distribution.

The mean and variance of ε are given by

$$E\left(\varepsilon \right)=\mu_{\varepsilon }=2\phi p+\lambda \left(1-\phi \right),$$

$$\mathrm{Var}\left(\varepsilon \right)=\sigma_{\varepsilon }^{2}=\lambda^{2}\left(1-\phi \right)\phi +2p\phi \left(1+p-2p\phi \right)+\lambda \left(1-\phi \right)\left(1-4p\phi \right).$$

The PGF of the innovation is given as

$$G_{\varepsilon }\left(s\right)=\phi {\left(1-p\right)}^{2}+2\phi p\left(1-p\right)s+s^{2}\phi p^{2}+\left(1-\phi \right)e^{-\lambda (1-s)}\;.$$

A distinctive structural merit of the proposed ZOTMPo distribution lies in the functional asymmetry of its probability weights. The linear mixing parameter ϕ governs the overall magnitude of the probability mass diverted from the baseline Poisson distribution, thereby ensuring a stable capacity for modification. In contrast, the allocation parameter *p* operates non-linearly, specifically quadratically, via ${\left(1-p\right)}^{2}$ and $p^{2}$, to distribute this diverted mass across the counts zero, one, and two. This non-linear formulation introduces a dynamic sensitivity, allowing the specific state inflation and deflation rates to accelerate or decelerate asymmetrically. Within the proposed INAR(1) framework, this quadratic allocation equips the innovation process with the mathematical flexibility required to capture the rapid, non-linear transitions between low-count states that are highly characteristic of early-stage epidemiological outbreaks.

**Figure 1:**
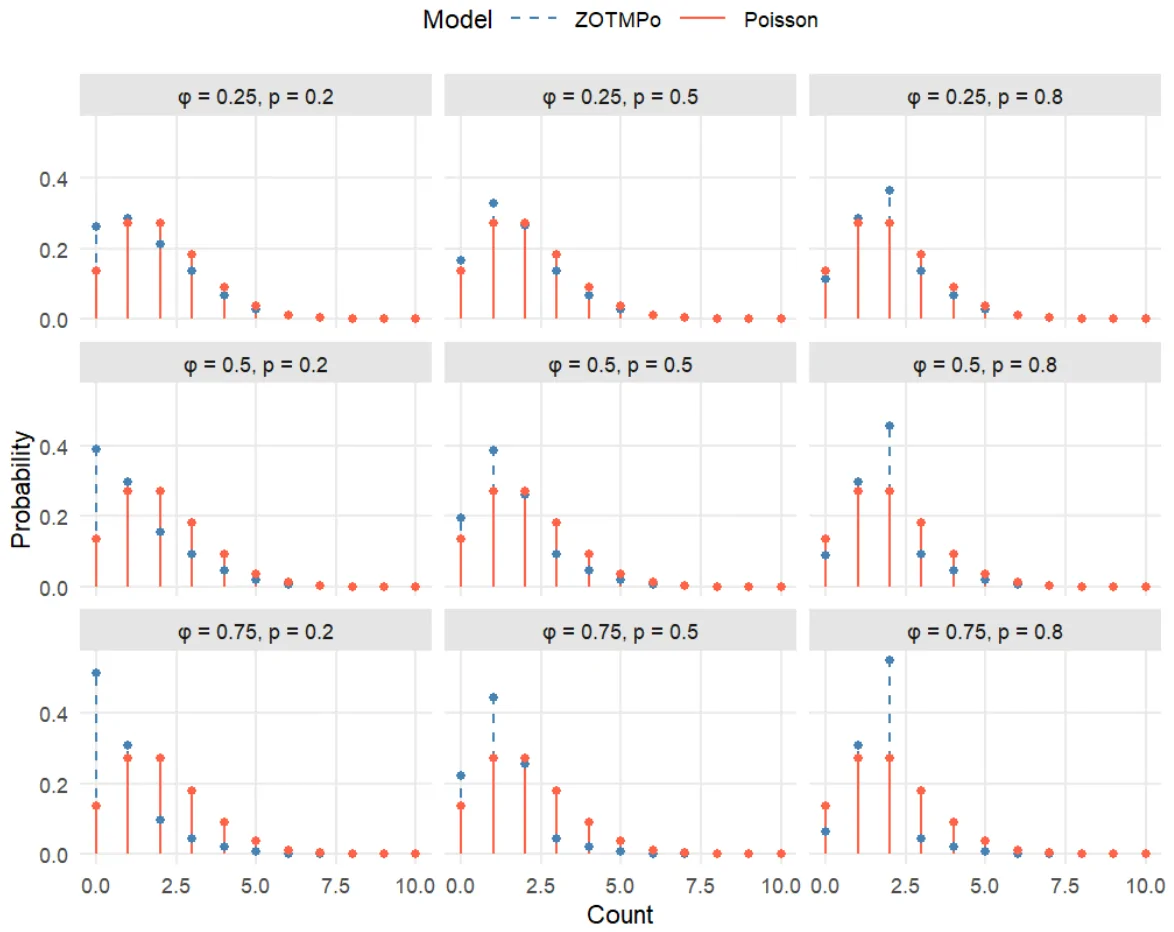
ZOTMPo distribution for various values of ϕ and *p*, with Poisson (λ=2).

Visually, Figure 1 displays the pmf of the ZOTMPo distribution for different choices of modification parameters ϕ and *p*, compared to the standard Poisson distribution with λ=2. The impact of the ZOTMPo modification is most evident at counts 0, 1, and 2. As ϕ increases, the probability mass is shifted away from the Poisson baseline and redistributed among these three categories according to *p*. Specifically, inflation or deflation at zero occurs through the term $\phi {\left(1-p\right)}^{2}$, at one through 2ϕp(1−p), and at two through $\phi p^{2}$. Consequently, we observe inflation of zeros when ϕ is large and *p* is small, inflation of ones when *p* is moderate, and inflation of twos when *p* is close to one. This flexible structure enables the model to capture unusual patterns of excess or deficit in the first three counts while retaining a Poisson-like tail for larger values. Inflation denotes the excess occurrence of the counts 0, 1, or 2 relative to that expected under a standard Poisson distribution, whereas deflation denotes their lower-than-expected occurrence. The proposed ZOTMPo distribution accommodates both inflation and deflation at these low-count values. Table 1 gives the conditions on parameters under which the three different inflation and deflation cases are observed.

**Figure 2:**
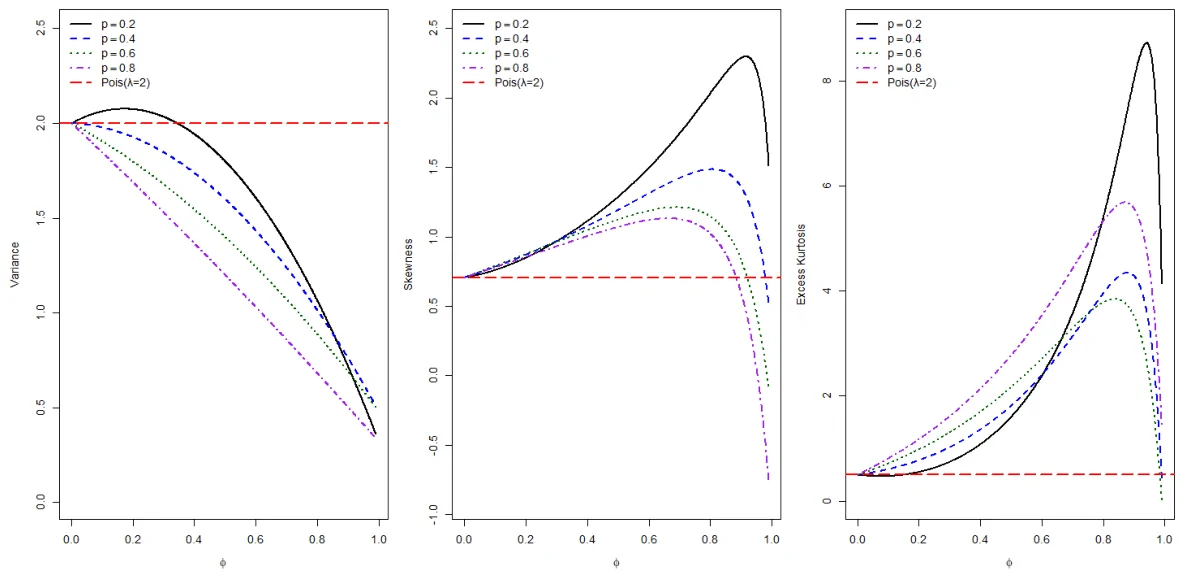
Variance, skewness, and kurtosis for different values of ϕ and *p*, with Poisson (λ=2).

Figure 2 illustrates the effect of the parameters ϕ and *p* on the variance, skewness, and excess kurtosis of the ZOTMPo distribution for λ=2, together with the corresponding Poisson moments. When ϕ=0, the ZOTMPo distribution coincides with the Poisson distribution; therefore, all three moments are identical to those of the Poisson distribution. As ϕ increases, the redistribution of probability mass among the counts 0, 1, and 2 produces systematic changes in the higher-order moments.

The variance varies with both ϕ and *p*, whereas the Poisson variance remains constant at λ=2. For smaller values of *p*, the variance remains close to the Poisson variance over a wider range of ϕ, while larger values of *p* result in a more rapid decrease in variance. The skewness and excess kurtosis also depart considerably from their Poisson counterparts. For moderate values of ϕ, both measures generally increase above the corresponding Poisson values, indicating greater asymmetry and heavier tails. As ϕ approaches one, the moments converge to those of the Bin(2,p) distribution because the Poisson component becomes negligible.

These results demonstrate that, for a fixed value of λ, the ZOTMPo distribution can produce a wide range of variance, skewness, and kurtosis values that are not attainable under the Poisson distribution. Consequently, the proposed distribution is suitable for count data exhibiting inflation or deflation at the lower counts together with departures from the Poisson moment characteristics.

## 3. Construction and Properties of the Process (ZOTMPo–INAR(1))

We introduce a general zero–one–two-modified Poisson INAR(1) process to handle stationary non-negative integer-valued time series. This model is based on the binomial thinning operator ‘∗’, defined as

$$\alpha *X=\sum_{i=1}^{X}W_{i}$$

where $W_{i}$ are i.i.d. Bernoulli random variables with parameter α and the probability generating function of *W* is $\Phi_{W}\left(\gamma \right)=\left(1-\alpha +\alpha \gamma \right),\left|\gamma \right|\leq 1.$ The random variables $\left\{W_{i}\right\}$ are mutually independent of X.

Now, we define the ZOTMPo–INAR(1) process to model stationary non-negative integer-valued time series data as follows.

**Definition** **1.** *A discrete-time stochastic process $\left\{X_{t}\right\}$ is said to follow a ZOTMPo–INAR(1) process if*

(3) $$X_{t}=\alpha *X_{t-1}+\varepsilon_{t},\;\;\;\;\;\alpha \in \left[0,1\right),\;\;\;\;\;t\in N,$$

*where*

*1.*  *$\left\{X_{t}\right\}$ is a sequence of non-negative integer-valued random variables,*
*2.*  *$\left\{\varepsilon_{t}\right\}$ is an i.i.d. sequence of non-negative integer-valued random variables following the ZOTMPo distribution,*
*3.*  *$\left\{\varepsilon_{t}\right\}$ is independent of $\left\{X_{t-1},X_{t-2},\ldots \right\}$ and of the thinning variables $\left\{W_{i}\right\}$.*

The unconditional mean and variance of $X_{t}$ are given by

$$\mu_{X}=E\left(X_{t}\right)=\frac{\mu_{\varepsilon }}{1-\alpha }=\frac{2\phi p+\lambda (1-\phi )}{1-\alpha },$$

$$\begin{array}{cc} \sigma_{X}^{2}=\mathrm{Var}\left(X_{t}\right) & =\frac{\alpha \mu_{\varepsilon }+\sigma_{\varepsilon }^{2}}{1-\alpha^{2}}=(\alpha (2\phi p+\lambda (1-\phi ))+\lambda^{2}\left(1-\phi \right)\phi +2p\phi \left(1+p-2p\phi \right)\\ & +\lambda \left(1-\phi \right)\left(1-4p\phi \right))/\left(1-\alpha^{2}\right). \end{array}$$

**Theorem** **1.** *Let $\left\{X_{t}\right\}$ be an INAR(1) process defined by (3). Then, under the condition α∈[0,1), the process $\left\{X_{t}\right\}$ has a unique strictly stationary and ergodic solution given by*

$$X_{t}\overset{d}{=}\sum_{j=0}^{\infty }\alpha^{j}*\varepsilon_{t-j}.$$

*The symbol $\overset{d}{=}$ denotes equality in distribution, $\alpha^{j}≜\underset{j\;\mathrm{times}}{\underset{︸}{\alpha *\alpha *\cdots *\alpha }}$, for j≥1, and $\alpha^{0}*\varepsilon ≜\varepsilon$. For all $t\in N_{0}$, the infinite sum is interpreted as the limit in probability of its corresponding finite sums.*

*The proof of this Theorem is provided in Appendix A.*

**Proposition** **1.** *The one-step-ahead conditional distribution of the process $\left\{X_{t}\right\}$ defined in (3) is given by*

P(Xt=y∣Xt−1=x) =P(α∗Xt−1+ε=y∣Xt−1=x) =∑k=0min{x,y}P(α∗Xt−1=k∣Xt−1=x)×P(ε=y−k) =∑k=0min{x,y}xkαk(1−α)x−k×P(ε=y−k), =∑k=0min{x,y}xkαk(1−α)x−k      ×ϕ(1−p)2+(1−ϕ)e−λ,if   y=k,2ϕp(1−p)+(1−ϕ)λe−λ,if   y=k+1,ϕp2+(1−ϕ)e−λλ22,if   y=k+2,(1−ϕ)e−λλ y−k(y−k)!,if   y≥k+3.

The one-step-ahead conditional mean and variance of the process $\left\{X_{t}\right\}$ are

$$\begin{array}{cc} E(X_{t}|X_{t-1}) & =\alpha X_{t-1}+\mu_{\varepsilon }\\ & =\alpha X_{t-1}+2\phi p+\lambda \left(1-\phi \right),\\ \mathrm{Var}(X_{t}|X_{t-1}) & =\alpha \left(1-\alpha \right)X_{t-1}+\sigma_{\varepsilon }^{2}\\ & =\alpha \left(1-\alpha \right)X_{t-1}+\lambda^{2}\left(1-\phi \right)\phi +2p\phi \left(1+p-2p\phi \right)+\lambda \left(1-\phi \right)\left(1-4p\phi \right). \end{array}$$

Using Equation (3) and recursion, we can express the *k*-step-ahead process as

(4) $$X_{t+k}=\alpha^{k}*X_{t}+\sum_{j=0}^{k-1}\alpha^{j}*\varepsilon_{t+k-j}.$$

The *k*-step-ahead mean and variance of the process are as follows.

$$\begin{array}{cc} E(X_{t+k}|X_{t}) & =\alpha^{k}X_{t}+\sum_{j=0}^{k-1}\alpha^{j}\mu_{\varepsilon }\\ & =\alpha^{k}X_{t}+\mu_{\varepsilon }\frac{\left(1-\alpha^{k}\right)}{\left(1-\alpha \right)},\;\;\;\;\;\left(\alpha \neq 1\right)\\ \mathrm{Var}(X_{t+k}|X_{t}) & =\alpha^{k}\left(1-\alpha^{k}\right)X_{t}+\sigma_{\varepsilon }^{2}\sum_{i=0}^{k-1}\alpha^{2i}+\alpha \;\mu_{\varepsilon }\left(\sum_{i=0}^{k-1}\alpha^{2i}-\alpha^{\;k-1}\frac{1-\alpha^{k}}{1-\alpha }\right)\\ & =\alpha^{k}\left(1-\alpha^{k}\right)X_{t}+\sigma_{\varepsilon }^{2}\frac{1-\alpha^{2k}}{1-\alpha^{2}}+\mu_{\varepsilon }\frac{\left(1-\alpha^{k}\right)\left(\alpha -\alpha^{k}\right)}{\left(1-\alpha^{2}\right)}. \end{array}$$

### Expected Run Lengths

The expected run length (ERL) of a specific value in a discrete distribution refers to the average number of observations between successive occurrences of that value. For the ZOTMPo distribution, the ERLs of 0, 1, and 2 indicate how frequently these values appear in the time series. Specifically, lower probabilities correspond to longer run lengths; that is, a value occurs less often. This measure helps us understand how often specific values, such as 0, 1, or 2, occur in sequence. It is useful for modeling real-world count data, in which values may occur more or less frequently than expected. ERLs are particularly valuable in applications such as surveillance, forecasting, or reliability analysis, as they quantify how the modified distribution differs from a standard Poisson process and help select models that better represent the observed data structure.

- Expected run length of zero:
  $$P_{00}=P\left(X_{t}=0|X_{t-1}=0\right)=\phi {\left(1-p\right)}^{2}+\left(1-\phi \right)e^{-\lambda }\;,$$
  and
  $$E\left[\mathrm{Run}\;\mathrm{length}\;\mathrm{of}\;\mathrm{zero}\right]=\frac{1}{1-P_{00}}=\frac{1}{1-\phi {\left(1-p\right)}^{2}-\left(1-\phi \right)e^{-\lambda }}\;.$$
- Expected run length of one:
  $$\begin{array}{cc} P_{11} & =P\left(X_{t}=1|X_{t-1}=1\right)=\alpha \left[\phi \left(1-p\right)\left(1-3p\right)+\left(1-\phi \right)e^{-\lambda }\left(1-\lambda \right)\right]\\ & \;+\left[2\phi p\left(1-p\right)+\left(1-\phi \right)\lambda e^{-\lambda }\right], \end{array}$$
  and
  $$E\left[\mathrm{Run}\;\mathrm{length}\;\mathrm{of}\;\mathrm{one}\right]=\frac{1}{1-P_{11}}.$$
- Expected run length of two:
  $$\begin{array}{cc} P_{22} & =P\left(X_{t}=2|X_{t-1}=2\right)={\left(1-\alpha \right)}^{2}\left[\phi p^{2}+\frac{\left(1-\phi \right)\lambda^{2}e^{-\lambda }}{2}\right]\\ & +2\alpha \left(1-\alpha \right)\left[2\phi p\left(1-p\right)+\left(1-\phi \right)\lambda e^{-\lambda }\right]+\alpha^{2}\left[\phi {\left(1-p\right)}^{2}+\left(1-\phi \right)e^{-\lambda }\right], \end{array}$$
  and
  $$E\left[\mathrm{Run}\;\mathrm{length}\;\mathrm{of}\;\mathrm{two}\right]=\frac{1}{1-P_{22}}.$$

The results presented in Table 2 demonstrate a remarkably close agreement between the theoretical and empirical expected run lengths for counts zero, one, and two, effectively validating the mathematical accuracy of the derived stochastic properties. Furthermore, the dynamic shifts in these run lengths across different parameter combinations highlight the model’s robust flexibility in capturing the highly variable frequencies of low-count occurrences inherent in real-world time series.

## 4. Information–Theoretic Properties of the ZOTMPo–INAR(1) Process

To assess the structural complexity and predictability of the proposed ZOTMPo–INAR(1) model, we examine its information-theoretic properties, specifically its Shannon entropy and conditional entropy. For integer-valued time series, entropy serves as a robust measure of uncertainty, effectively capturing the complex fluctuations introduced by both the thinning operation and the modified innovation sequence.

### 4.1. Entropy of the ZOTMPo Innovation Sequence

The innovation process, $\left\{\varepsilon_{t}\right\}$, is the primary source of new uncertainty injected into the system at each time step *t*. Given that $\varepsilon_{t}∼\mathrm{ZOTMPo}\left(\phi ,\lambda ,p\right)$, its marginal Shannon entropy is defined as

$$H\left(\varepsilon_{t}\right)=-\sum_{i=0}^{\infty }P\left(\varepsilon_{t}=i\right)\log P\left(\varepsilon_{t}=i\right).$$

Using the definition of the ZOTMPo probability mass function (PMF), an exact analytical expression for H(ε) can be derived by decomposing the sum into its low-count states (i=0,1,2) and the infinite Poisson tail (i≥3). This yields

$$H\left(\varepsilon_{t}\right)=-\sum_{i=0}^{2}P\left(\varepsilon_{t}=i\right)\log P\left(\varepsilon_{t}=i\right)-\sum_{i=3}^{\infty }\left[\left(1-\phi \right)\frac{\lambda^{i}e^{-\lambda }}{i!}\right]\log\left[\left(1-\phi \right)\frac{\lambda^{i}e^{-\lambda }}{i!}\right].$$

This formulation explicitly shows how the mixing parameter and the quadratic allocation parameter *p* modulate the innovation entropy. Depending on whether the structural modification causes inflation or deflation at low counts, $H(\varepsilon_{t})$ will systematically deviate from the entropy of a standard Poisson random variable.

### 4.2. Conditional Entropy of the Markov Process

As the ZOTMPo–INAR(1) model satisfies the first-order Markov property, its complexity can be characterized by its conditional entropy. Let $P_{x,y}=P\left(X_{t}=y|X_{t-1}=x\right)$ denote the one-step transition probability. The conditional entropy of $X_{t}$ given the previous state $X_{t-1}=x$ is

$$H\left(X_{t}|X_{t-1}=x\right)=-\sum_{y=0}^{\infty }P_{x,y}\log P_{x,y}.$$

The transition probability $P_{x,y}$ is the convolution of the binomial thinning operator and the ZOTMPo innovation. Using the innovation’s PMF from Equation (2), it is given explicitly by

Px,y=∑k=0min{x,y}xkαk(1−α)x−kP(εt=y−k).

### 4.3. Entropy Bounds and Structural Dynamics

To theoretically bound the uncertainty of the ZOTMPo–INAR(1) model, we employ the fundamental properties of Shannon entropy for independent random variables. Given that $X_{t}=\alpha *X_{t-1}+\varepsilon_{t}$ and conditioning on $X_{t-1}=x$, the binomial survivor component $B_{t}=\alpha *X_{t-1}$ is independent of the innovation $\varepsilon_{t}$ conditional on $X_{t-1}$. Consequently, for a given state *x*, the entropy of the convolution satisfies the following inequality:

$$\max\left\{H\left(\mathrm{Bin}\left(x,\alpha \right)\right),H\left(\varepsilon_{t}\right)\right\}\leq H\left(X_{t}|X_{t-1}=x\right)\leq H\left(\mathrm{Bin}\left(x,\alpha \right)\right)+H\left(\varepsilon_{t}\right),$$

where H(Bin(x,α)) is the entropy of a binomial random variable. This inequality provides an insight into the model’s dynamics: the total uncertainty of the next state, $X_{t}$, is bounded from below by the complexity of the ZOTMPo innovation, and from above by the sum of uncertainties from both the disease survival (thinning) and new infections (innovations).

#### Proof of the Entropy Bounds

Let $X_{t}$ be the state of the ZOTMPo–INAR(1) process at time *t*. Conditioned on $X_{t-1}=x$, the conditional distribution of the process is equivalent to the sum of two mutually independent discrete random variables:

$$X_{t}|\left(X_{t-1}=x\right)∼B_{t}+\varepsilon_{t},$$

where $B_{t}∼\mathrm{Bin}\left(x,\alpha \right)$ represents the survival process, and $\varepsilon_{t}∼\mathrm{ZOTMPo}\left(\phi ,\lambda ,p\right)$ represents the innovation. We aim to bound the conditional entropy $H\left(X_{t}|X_{t-1}=x\right)=H\left(B_{t}+\varepsilon_{t}\right)$.

For the upper bound, by the fundamental properties of Shannon entropy, the entropy of the sum of independent random variables is bounded by their joint entropy. Since $B_{t}$ and $\varepsilon_{t}$ are independent, their joint entropy equals the sum of their marginal entropies:

$$H\left(B_{t}+\varepsilon_{t}\right)\leq H\left(B_{t},\varepsilon_{t}\right)=H\left(B_{t}\right)+H\left(\varepsilon_{t}\right).$$

Therefore,

$$H\left(X_{t}|X_{t-1}=x\right)\leq H\left(\mathrm{Bin}\left(x,\alpha \right)\right)+H\left(\varepsilon_{t}\right).$$

This implies that the total uncertainty of the next state cannot exceed the combined uncertainties of the thinning operation and the innovations.

For the lower bound, adding an independent random variable acts as a “smoothing” operation that never decreases uncertainty. Thus, the entropy of a sum of independent variables is at least as large as the entropy of any single component:

$$H\left(B_{t}+\varepsilon_{t}\right)\geq H\left(B_{t}\right)\;\mathrm{and}\;H\left(B_{t}+\varepsilon_{t}\right)\geq H\left(\varepsilon_{t}\right).$$

This directly implies:

$$H\left(B_{t}+\varepsilon_{t}\right)\geq \max\left\{H\left(B_{t}\right),H\left(\varepsilon_{t}\right)\right\}.$$

Therefore,

$$H\left(X_{t}|X_{t-1}=x\right)\geq \max\left\{H\left(\mathrm{Bin}\left(x,\alpha \right)\right),H\left(\varepsilon_{t}\right)\right\}.$$

This establishes that the overall process is always at least as unpredictable as its most chaotic underlying component, completing the proof.

As the autoregressive parameter α→0 (no serial dependence), we have H(Bin(x,α))→0, and the marginal entropy of the process converges exactly to the marginal entropy of the ZOTMPo innovation, i.e., $H\left(X_{t}\right)\to H\left(\varepsilon_{t}\right)$. Conversely, as α→1, the structural memory of the process becomes dominant, and the entropy reflects the full propagation of historical variance, continuously supplemented by the modified low-count noise.

## 5. Estimation of Parameters

In this section, a method for estimating the parameters of the ZOTMPo–INAR(1) process is discussed: the conditional maximum likelihood (CML) method.

### 5.1. Conditional Maximum Likelihood Estimation (CML)

The CML estimator of θ=(α,ϕ,λ,p) is the value $\theta_{CML}$ that maximizes the likelihood. Since the likelihood is a non-linear function, the maximum likelihood estimate of θ is computed using numerical methods. The CML estimates of the parameters can be obtained by maximizing the log-likelihood function,

(5) $$\log L\left(x_{1},\ldots ,x_{n};\theta \right)=\sum_{t=2}^{n}\log P\left(x_{t}|x_{t-1}\right).$$

### 5.2. Monte Carlo Simulation Study

A simulation study was conducted to assess the performance of the proposed estimation procedure. We generated 1000 simulations of the process for sample sizes n=50,100,500, and 1000, using three different parameter combinations: θ=(0.2,0.7,4,0.3), θ=(0.8,0.5,3,0.7), and θ=(0.2,0.1,7,0.1). Parameter estimates were obtained using the conditional maximum likelihood (CML) method, as conditional least squares (CLS) and Yule–Walker (YW) approaches did not provide stable or accurate results.

**Figure A1:**
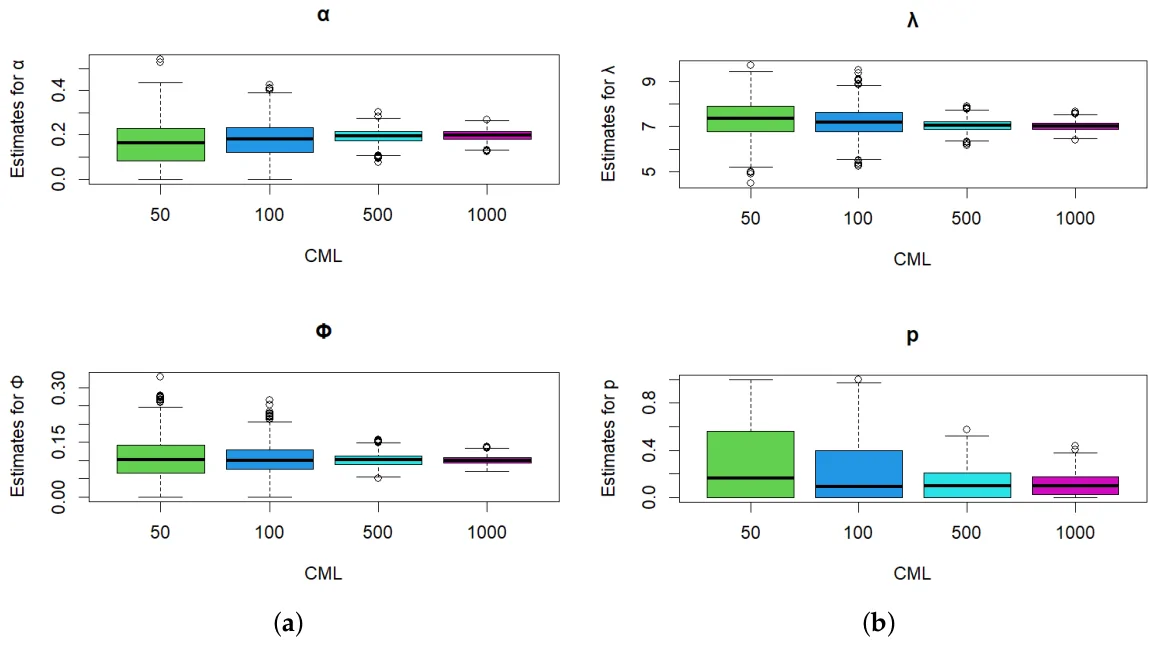
Boxplots of the CML estimates for α=0.2, ϕ=0.1, λ=7, p=0.1: (**a**) estimates of α and ϕ; (**b**) estimates of λ and *p*.

**Figure A2:**
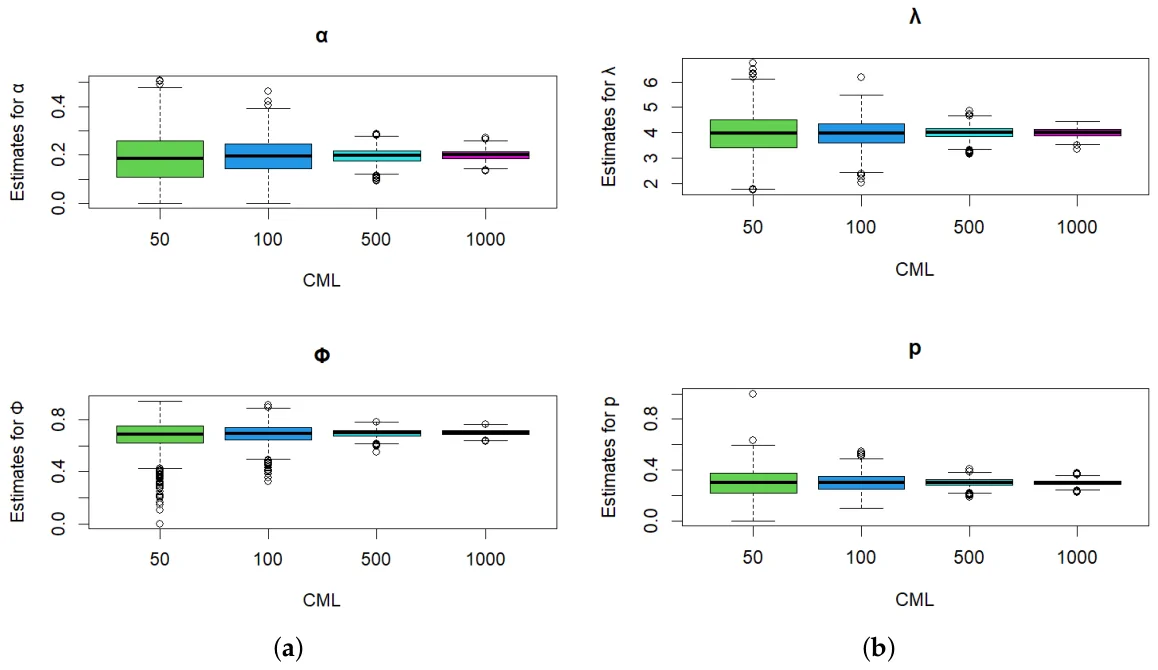
Boxplots of the CML estimates for α=0.2, ϕ=0.7, λ=4, p=0.3: (**a**) estimates of α and ϕ; (**b**) estimates of λ and *p*.

**Figure A3:**
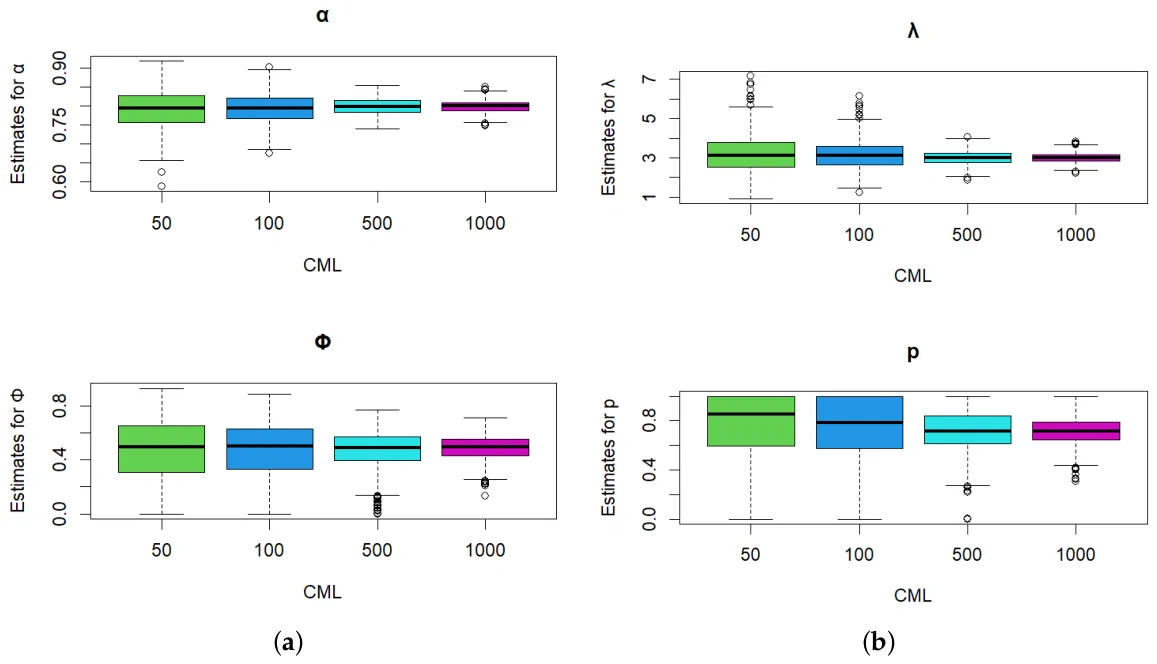
Boxplots of the CML estimates for α=0.8, ϕ=0.5, λ=3, p=0.7: (**a**) estimates of α and ϕ; (**b**) estimates of λ and *p*.

**Figure A4:**
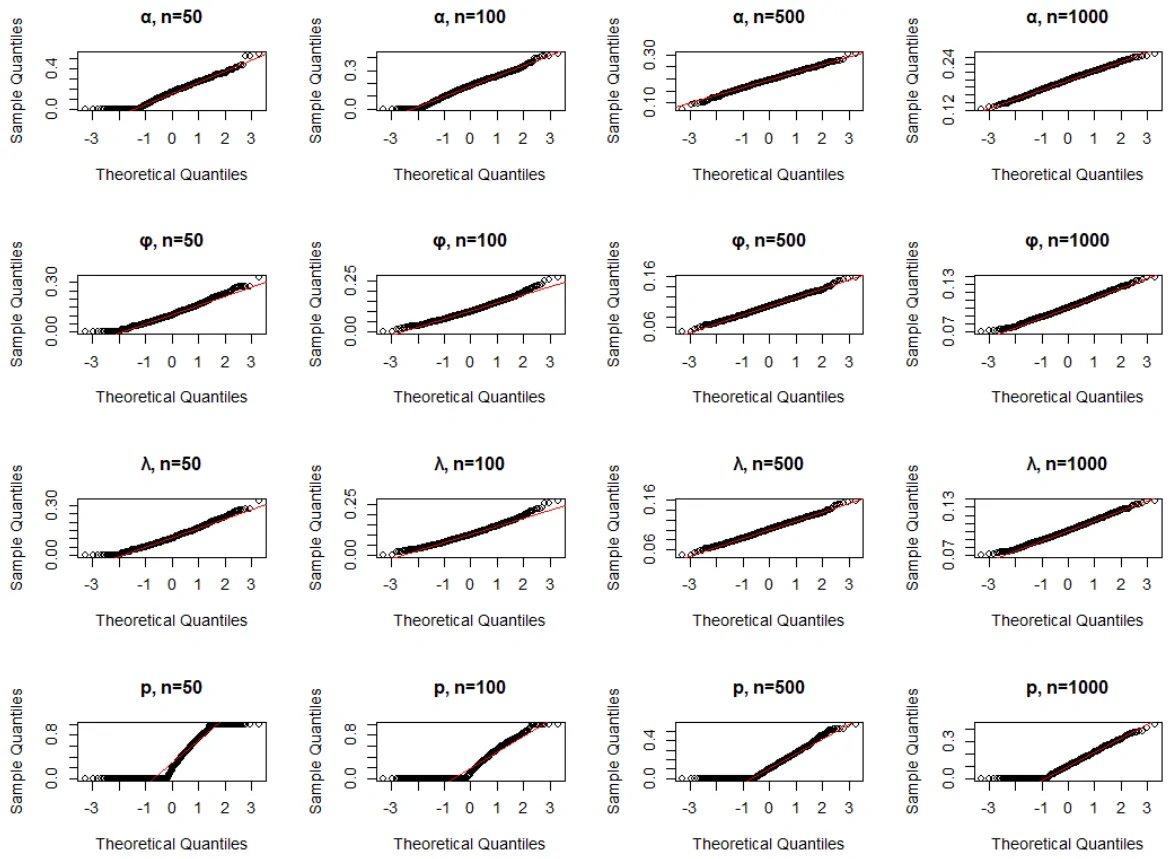
Q–Q plot for α=0.2, ϕ=0.1, λ=7, p=0.1.

The mean and mean squared error (MSE) of the CML estimates across all simulations are reported in Table 3. For smaller sample sizes (e.g., n=50), some parameters, particularly *p*, exhibit slight small-sample bias. However, this bias rapidly vanishes as *n* increases, confirming the consistency of the CML estimators. To visually assess the convergence of the estimators, boxplots for three parameters across different sample sizes are shown in Figure A1, Figure A2 and Figure A3. These plots show that the estimates converge to the true parameter values as the sample size increases. The Q–Q plots in Figure A4 for sample sizes 50, 100, 500, and 1000 suggest that the normality assumption holds, with the fit improving as the sample size increases.

## 6. Data Analysis

### 6.1. Model Adequacy and Diagnostic Analysis of Considered Datasets

Model validation is an essential step in the analysis of integer-valued autoregressive processes, particularly for count data exhibiting overdispersion and excess low counts. After estimating the model parameters via CML, diagnostic checks such as the cumulative periodogram and a jump process were employed to assess whether the fitted model adequately captures both the serial dependence structure and the distributional characteristics of the observed data.

The diagnostic framework includes time-domain residual analysis, frequency-domain investigation through the cumulative periodogram, jump process analysis, and goodness-of-fit measures. These complementary tools provide a comprehensive assessment of the model’s adequacy by examining residual independence, structural stability, and predictive performance [19].

**Pearson Residual Diagnostics**

Let ${\left\{X_{t}\right\}}_{t=1}^{n}$ denote the observed count process. The standardized Pearson residuals are defined as

(6) $$r_{t}=\frac{X_{t}-E\left(X_{t}|X_{t-1}\right)}{\sqrt{\mathrm{Var}(X_{t}|X_{t-1})}},\;\;\;\;\;t=2,\ldots ,n,$$

where the conditional mean and variance are computed under the fitted ZOTMPo–INAR(1) model, with unknown parameters replaced by their maximum likelihood estimates.

Under the correct model specification, the residual sequence $\left\{r_{t}\right\}$ should behave approximately as an uncorrelated process with zero mean and unit variance. The residual sample autocorrelation function (ACF) is examined to detect any remaining serial dependence.

For the fitted model, all residual autocorrelations lie within the 95% confidence bounds, indicating that the autoregressive structure has been appropriately specified and that no systematic serial pattern remains in the residual sequence.

**Cumulative Periodogram of Residuals**

To complement the time-domain diagnostics, a frequency-domain assessment is conducted using the cumulative periodogram of the Pearson residuals. This diagnostic is effective for validating INAR-type count processes [19].

If the model is correctly specified, the cumulative periodogram should fluctuate randomly around the 45° reference line and remain within the corresponding Kolmogorov–Smirnov confidence bands. The empirical cumulative periodogram obtained from the fitted ZOTMPo–INAR(1) model does not exhibit systematic deviations from this benchmark, providing further evidence that the residuals behave approximately as white noise.

**Jump Process Analysis**

Additional insight into the dynamic adequacy of the model is obtained through the jump process

$$J_{t}=X_{t}-X_{t-1},\;\;\;\;\;t\geq 2.$$

Jump-based diagnostics are particularly informative for INAR-type models, as they reflect both the thinning-induced persistence and the innovation-driven variability [19]. For a stationary INAR(1)-type process, the jump variance satisfies

$$\mathrm{Var}\left(J_{t}\right)=2\left[\gamma_{X}\left(0\right)-\gamma_{X}\left(1\right)\right],$$

where $\gamma_{X}\left(0\right)=\mathrm{Var}\left(X_{t}\right)$ and $\gamma_{X}\left(1\right)=\mathrm{Cov}\left(X_{t},X_{t-1}\right)$.

Using the autoregressive covariance structure $\gamma_{X}\left(1\right)=\rho \gamma_{X}\left(0\right),$ where ρ=α is the autoregressive parameter of the INAR(1) model, we obtain

$$\mathrm{Var}\left(J_{t}\right)=2\left(1-\rho \right)\gamma_{X}\left(0\right),$$

which is evaluated at the estimated parameter values.

A Shewhart-type control chart is constructed for $\left\{J_{t}\right\}$ using control limits $\pm 3\sqrt{\mathrm{Var}(J_{t})}$. The resulting plot shows that the observed jumps remain within the prescribed bounds, with no abnormal excursions or clustering behavior. This indicates that the fitted ZOTMPo–INAR(1) model successfully captures variability.

**Ljung–Box test**

The presence of autocorrelation in a time series can be assessed using the Ljung–Box test [19]. This test examines whether the autocorrelations of the residuals are significantly different from zero. If the associated *p*-values exceed the chosen significance level (typically 0.05), the null hypothesis of no autocorrelation is not rejected, indicating that the residuals are independently distributed. This, in turn, suggests that the fitted model adequately captures the underlying dependence structure in the data.

**Scoring Rules**

Several scoring rules have been proposed to evaluate the accuracy of predictive distributions [19]. In general, a scoring rule is defined as $s\left(p\left(\cdot |x_{t-1}\right),\;x_{t}\right),$ where the observed value $x_{t}$ is evaluated against the one-step-ahead predictive distribution $p(\cdot |x_{t-1})$. The associated predictive cumulative distribution function is given by $F_{k|x_{t-1}}=\sum_{j=0}^{k}p_{j|x_{t-1}}.$ Smaller scores indicate better predictive performance.

For a time series $x_{1},\ldots ,x_{T}$, the overall predictive accuracy is assessed using the mean score

$$\frac{1}{T-1}\sum_{t=2}^{T}s\left(p\left(\cdot |x_{t-1}\right),\;x_{t}\right).$$

As in [19], we used three proper scoring rules: the logarithmic, quadratic, and ranked probability scores. These measures provide a framework for comparing competing models in terms of probabilistic forecast performance.

Since the model is first-order Markov, the scoring rules are computed for t=2,…,T, and the reported values correspond to the average of the (T−1) one-step-ahead predictions.

Logarithmic Score (LS): The logarithmic score at time *t* is defined as

$$s_{ls}\left(t\right)=-\log p_{x_{t}|x_{t-1}},$$

and the mean logarithmic score is given by

(7) $$Mean_{LS}=\frac{1}{T-1}\sum_{t=2}^{T}\left(-\log p_{x_{t}|x_{t-1}}\right).$$

Quadratic Score (QS): The quadratic score is defined as

$$s_{qs}\left(t\right)=-2p_{x_{t}|x_{t-1}}+\sum_{k=0}^{\infty }p_{k|x_{t-1}}^{2},$$

and the corresponding mean quadratic score is

(8) $$Mean_{QS}=\frac{1}{T-1}\sum_{t=2}^{T}\left(-2p_{x_{t}|x_{t-1}}+\sum_{k=0}^{\infty }p_{k|x_{t-1}}^{2}\right).$$

Ranked Probability Score (RPS): The ranked probability score at time *t* is defined as

$$s_{rps}\left(t\right)=\sum_{k=0}^{\infty }{\left(F_{k|x_{t-1}}-1\left(X_{t}\leq k\right)\right)}^{2},$$

where

$$1\left(X_{t}\leq k\right)=\left\{\begin{array}{cc} 1, & \mathrm{if}\;X_{t}\leq k,\\ 0, & \mathrm{otherwise}. \end{array}\right.$$

and its average value is

(9) $$Mean_{RPS}=\frac{1}{T-1}\sum_{t=2}^{T}\sum_{k=0}^{\infty }{\left(F_{k|x_{t-1}}-1\left(X_{t}\leq k\right)\right)}^{2}.$$

Information Criteria and Forecast Error Measures: Model comparison is additionally supported using the information criteria

(10) AIC=−2ℓ+2k,     BIC=−2ℓ+klog(T),

where *ℓ* denotes the conditional log-likelihood, and *k* is the number of estimated parameters.

Other goodness-of-fit (GOF) measures used to assess the fit of the model to the data are the root mean square error (RMSE) and mean absolute error (MAE):

(11) $$RMSE=\sqrt{\frac{1}{T-1}\sum_{t=2}^{T}{\left(x_{t}-{\hat{x}}_{t}\right)}^{2}},\;\;\;\;\;MAE=\frac{1}{T-1}\sum_{t=2}^{T}\left|x_{t}-{\hat{x}}_{t}\right|,$$

where ${\hat{x}}_{t}=E\left(X_{t}|X_{t-1}=x_{t-1}\right)$ denotes the one-step conditional mean forecast.

The model parameters were estimated from the observed data using conditional maximum likelihood (CML). Model comparison was performed using the Akaike Information Criterion (AIC), Bayesian Information Criterion (BIC), root mean square error (RMSE), mean absolute error (MAE), logarithmic score (LS), quadratic score (QS), and ranked probability score (RPS). AIC and BIC measure model fit with a penalty for the number of parameters, where BIC applies a stronger penalty. RMSE and MAE assess point forecast accuracy, while LS, QS, and RPS evaluate the full predictive distribution. For all criteria, smaller values indicate better performance.

### 6.2. Dengue Fever Cases

We analyze weekly counts of dengue fever cases reported in Bavaria, Germany, for the period 2013–2016. The dataset, obtained from the Robert Koch Institute (https://survstat.rki.de, accessed on 19 January 2026), consists of 167 observations.

**Figure 3:**
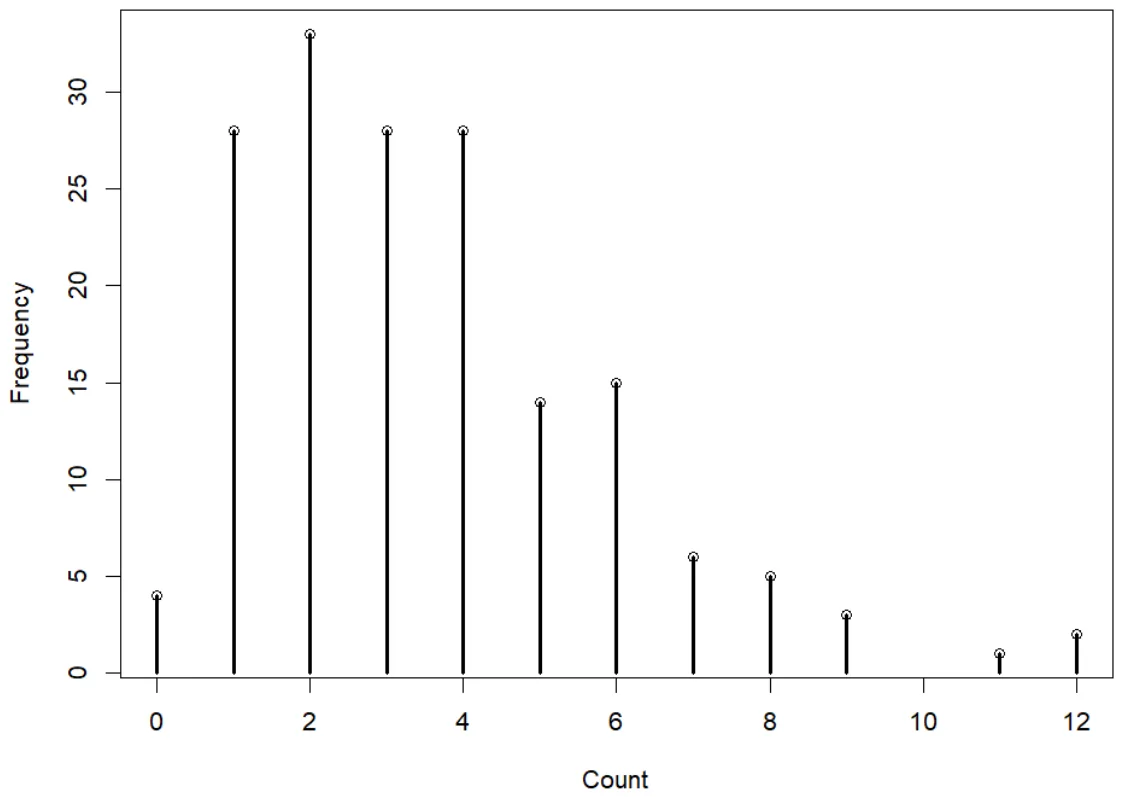
Frequency plot of counts for dengue dataset.

**Figure 4:**
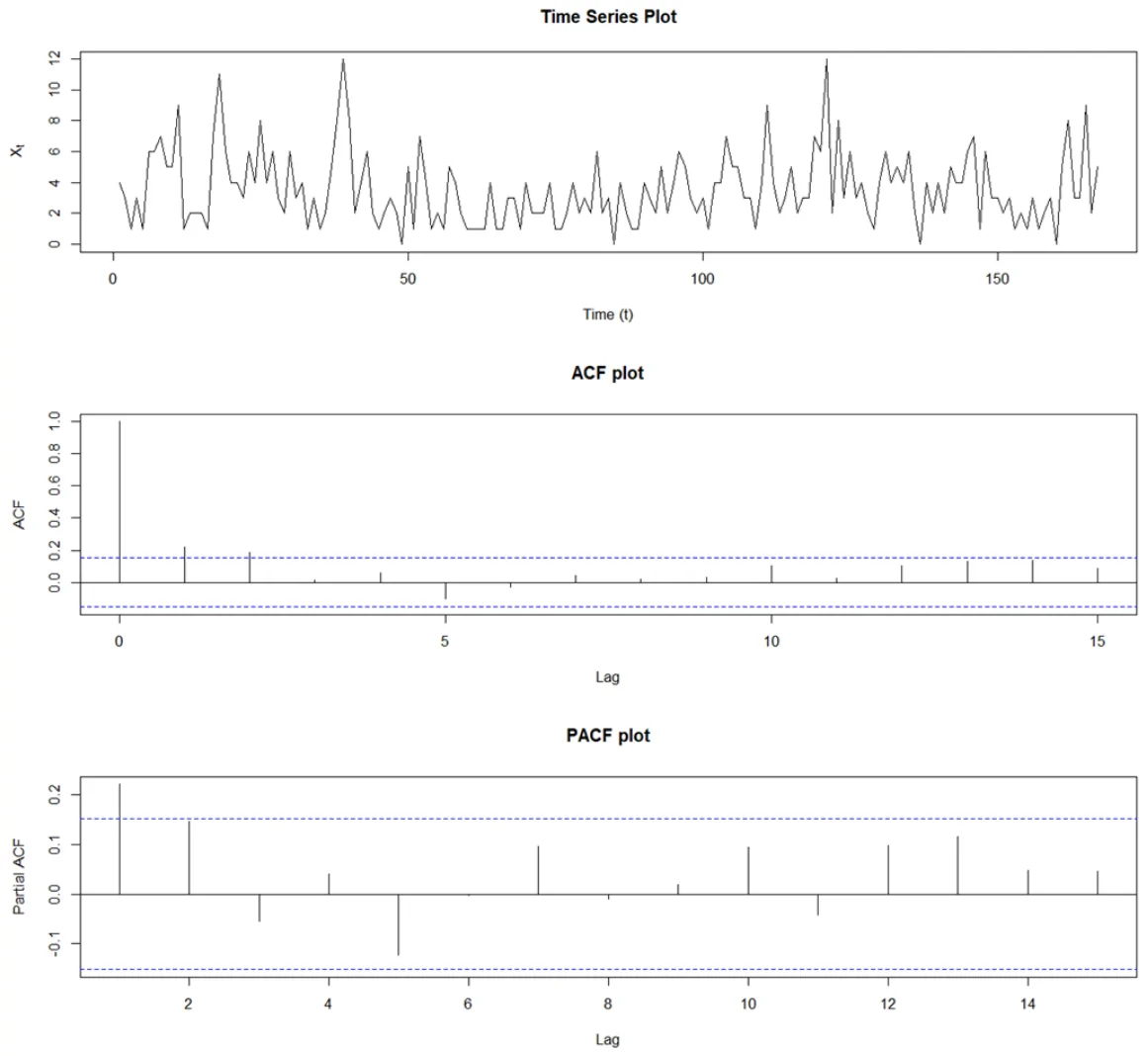
Sample path, sample ACF, and sample PACF plots for the dengue dataset.

The frequency distribution of the data, shown in Figure 3, indicates inflation at the value 2. The sample mean and variance are 3.5569 and 5.4410, respectively, indicating overdispersion. The time series plot together with the sample ACF and PACF (Figure 4) does not exhibit any clear seasonal pattern, suggesting a non-seasonal structure.

The Euclidean distances between the actual and estimated probabilities for counts zero, one, and two under the geometric, Poisson, TGD, ZMG, and ZOTMPo marginal distributions are reported in Table 4. Among the models considered, the ZOTMPo distribution has the smallest Euclidean distance, indicating the best approximation to the observed probabilities.

Parameter estimates and model comparison criteria that include AIC, BIC, RMSE, MAE, and various scoring functions for competing INAR(1) models are summarized in Table 5. For the fitted ZOINBINAR(1) process, the estimated zero-inflation parameter is $\hat{\alpha }=10^{-8}$, which is reported as 0.0000 after rounding to four decimal places. This indicates that the zero-inflation component contributes negligibly to the fitted ZOINBINAR(1) process. The results indicate that the ZOTMPo–INAR(1) model demonstrates highly competitive performance, outperforming the alternative models on several key metrics (AIC, BIC, MAE, etc.), while maintaining strong theoretical consistency with the underlying low-count dynamics.

**Figure 5:**
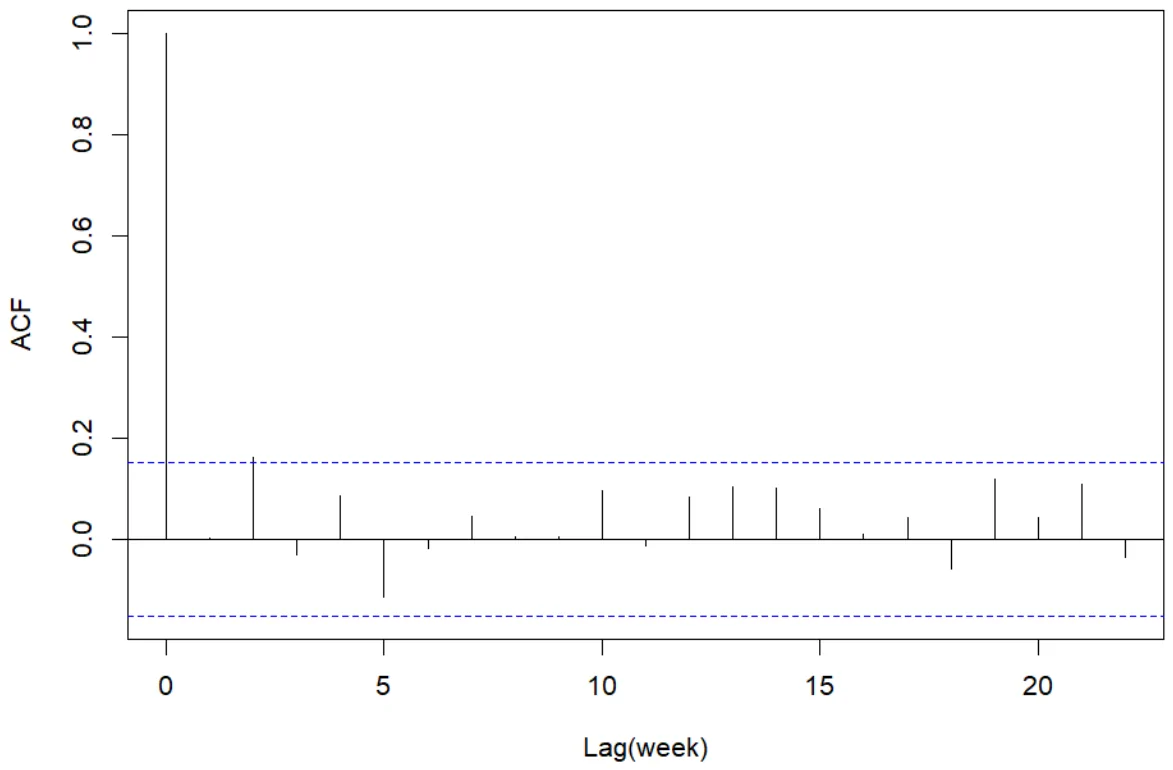
Sample ACF of Pearson residuals for dengue dataset.

**Figure 6:**
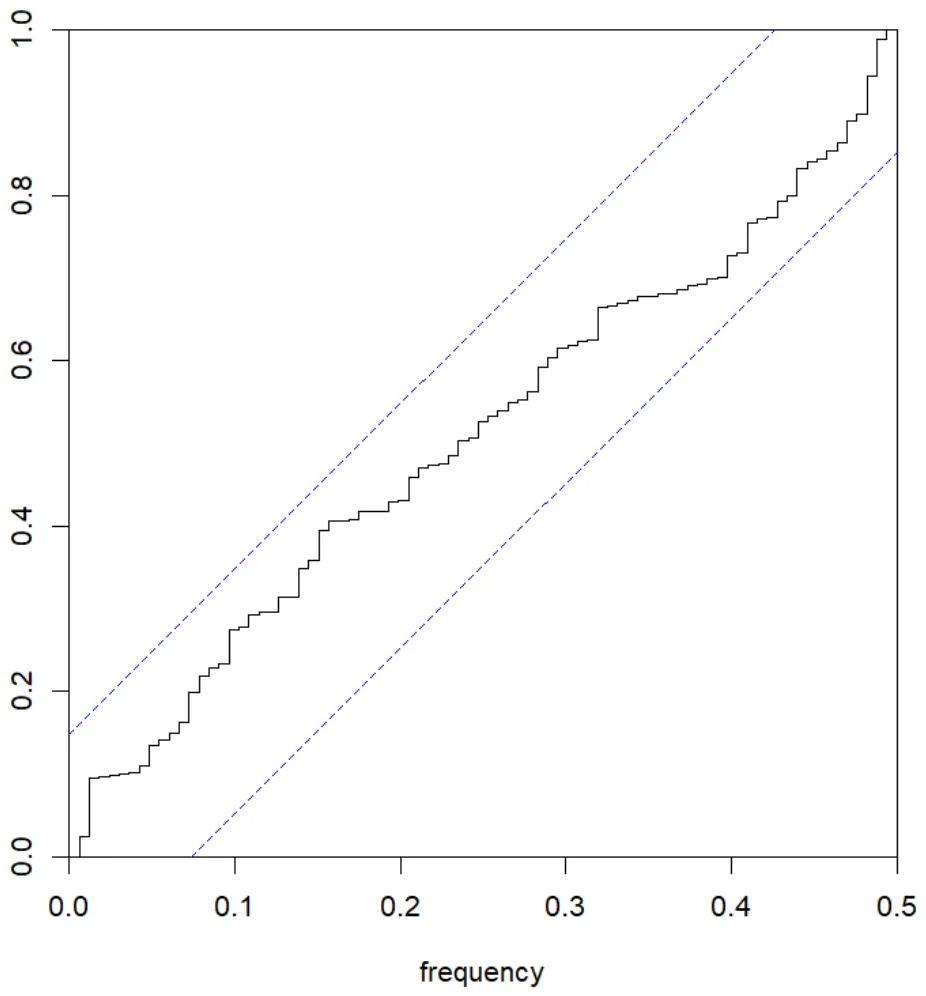
The cumulative periodogram plot of the Pearson residuals for the dengue dataset.

The adequacy of the fitted model is further supported by the residual diagnostics. As shown in Figure 5, the autocorrelation function (ACF) of Pearson residuals does not exhibit significant autocorrelation. Similarly, the cumulative periodogram in Figure 6 is entirely within the confidence limits of 95% and closely follows the expected diagonal pattern, indicating no significant departure from white noise. Together, these results suggest the absence of residual autocorrelation structure, confirming that the model adequately captures the temporal dynamics of the dengue dataset.

**Figure 7:**
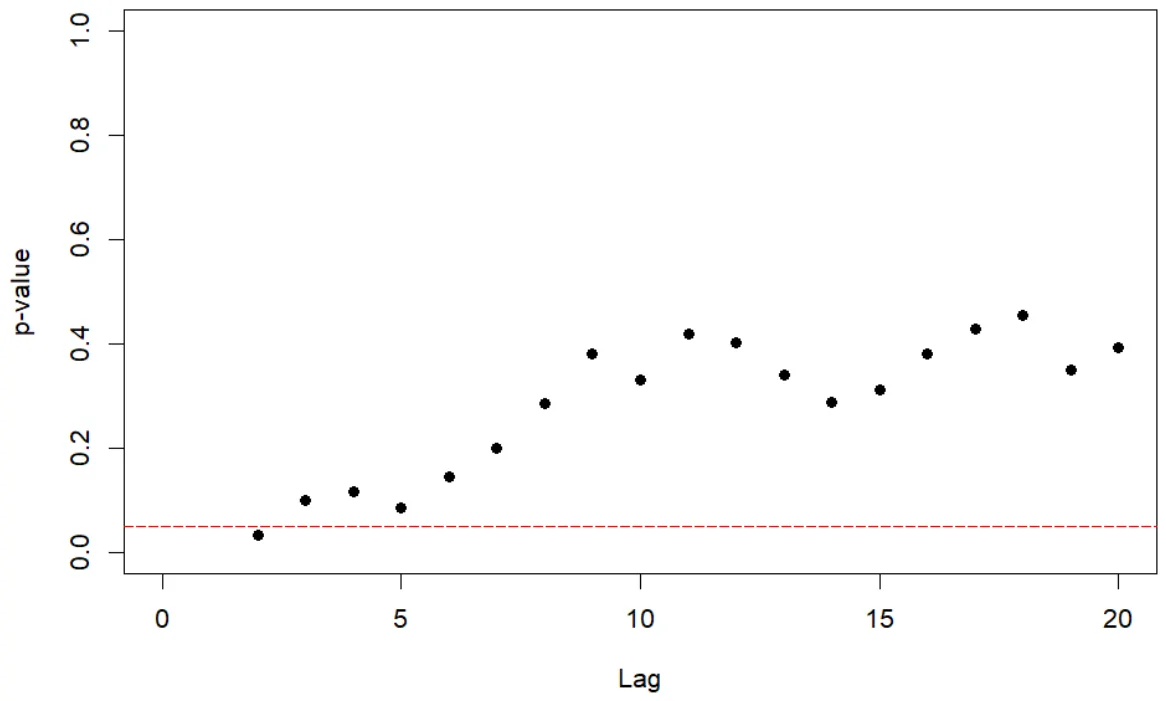
The Ljung–Box *p*–values for the dengue dataset.

**Figure 8:**
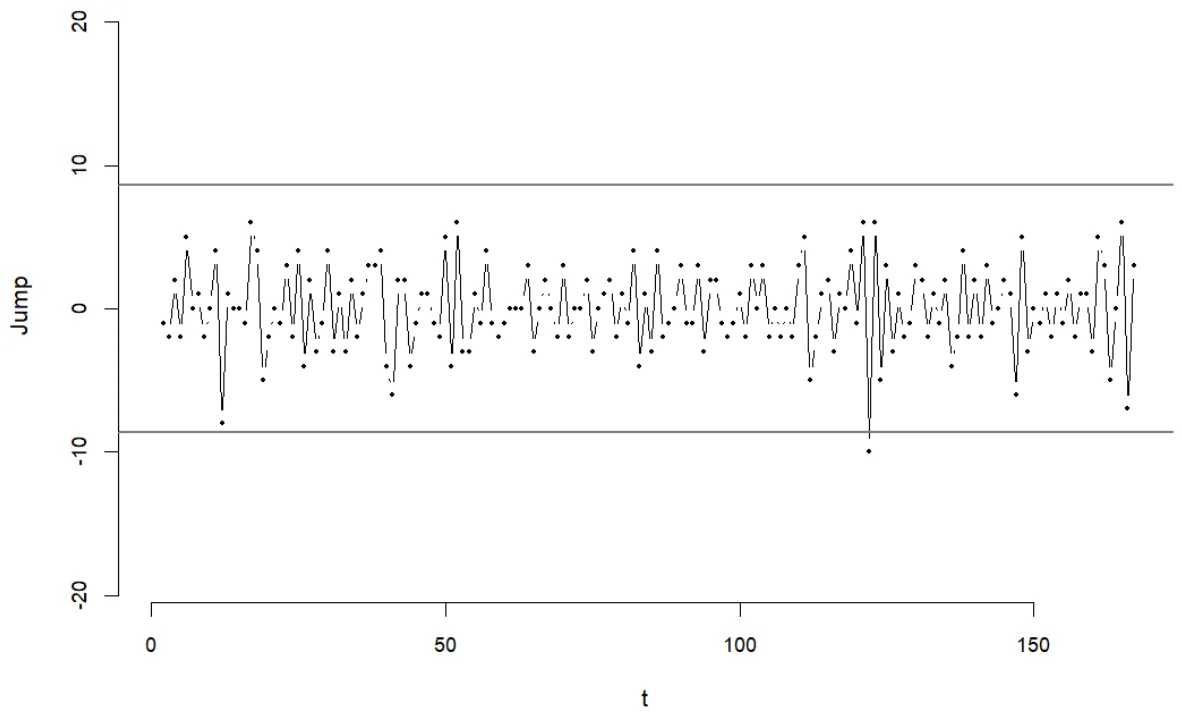
The jumps against time of the proposed model for the dengue dataset.

Additional diagnostic checks reinforce this conclusion. The Ljung–Box test *p*-values (Figure 7) are predominantly above the 0.05 significance level, which provides no strong evidence against the null hypothesis of independence, although a marginal deviation at lower lags suggests weak short-term dependence. Furthermore, the jump process (Figure 8) fluctuates around zero, with most observations falling within the control limits and only a few isolated spikes, indicating occasional abrupt changes, but no systematic pattern.

**Figure 9:**
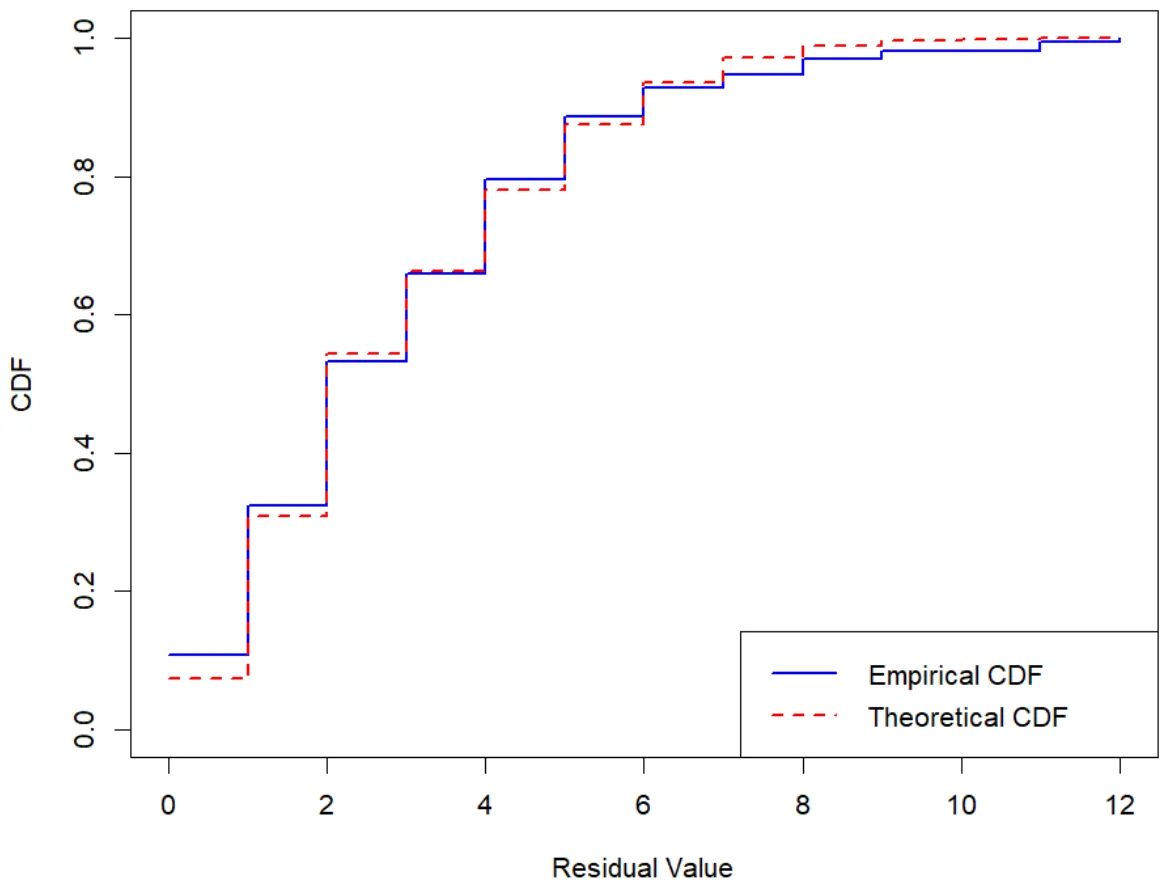
Empirical vs. theoretical CDF of ZOTMPo distribution for the dengue dataset.

The last 10 observations in the dataset were used for forecasting. The multi-step-ahead forecasts derived from the fitted model are presented in Table 6. Finally, the comparison between the empirical and theoretical cumulative distribution functions in Figure 9 demonstrates that the fitted ZOTMPo innovation distribution provides an adequate representation of the dengue data.

**Comparative Models:**

- PoINAR(1): The INAR(1) process with Poisson marginal distributions, introduced based on a binomial thinning operator [20].
- NGINAR(1): The INAR(1) process with new geometric marginal distributions, introduced based on a negative binomial thinning operator [21].
- GINAR(1): The INAR(1) process with a geometric marginal distribution, introduced based on a binomial thinning operator [22].
- ZMGINAR(1): The INAR(1) process with zero-modified geometric marginal distributions, introduced based on a negative binomial thinning operator [4].
- TGDINAR(1): The INAR(1) process with transmuted geometric marginal distributions, introduced based on a binomial thinning operator [23].
- ZOINBINAR(1): The INAR(1) process with zero-one inflated negative binomial innovation distribution based on a binomial thinning operator [24].
- ZOTMPo–INAR(1): The proposed model.

### 6.3. E. coli Enteritis

We analyze weekly counts of *E. coli* enteritis cases reported in the Territorial Unit Stuttgart, Baden-Württemberg, Germany, from 2001 to 2005. The dataset, obtained from the Robert Koch Institute (https://survstat.rki.de, accessed on 25 January 2026), contains 260 observations.

**Figure 10:**
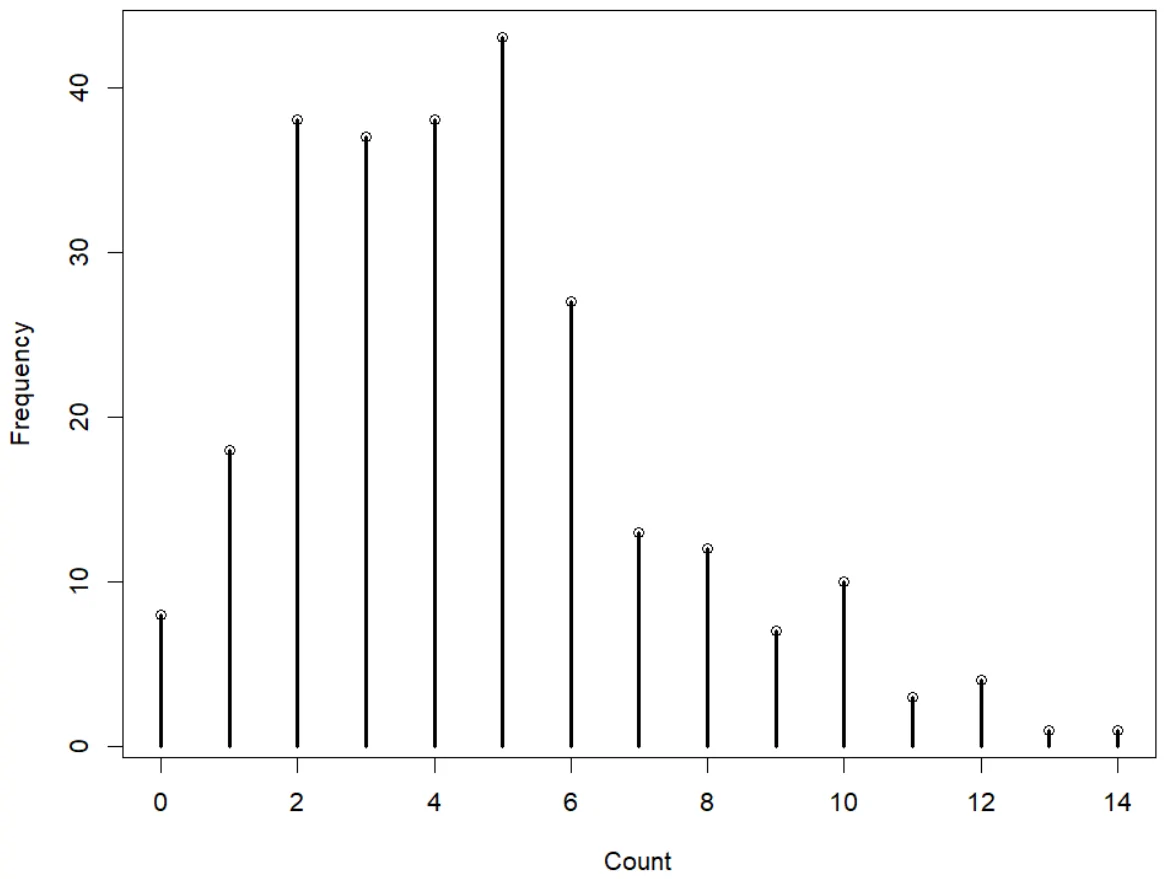
Frequency plot of counts for *E. coli* enteritis cases.

**Figure 11:**
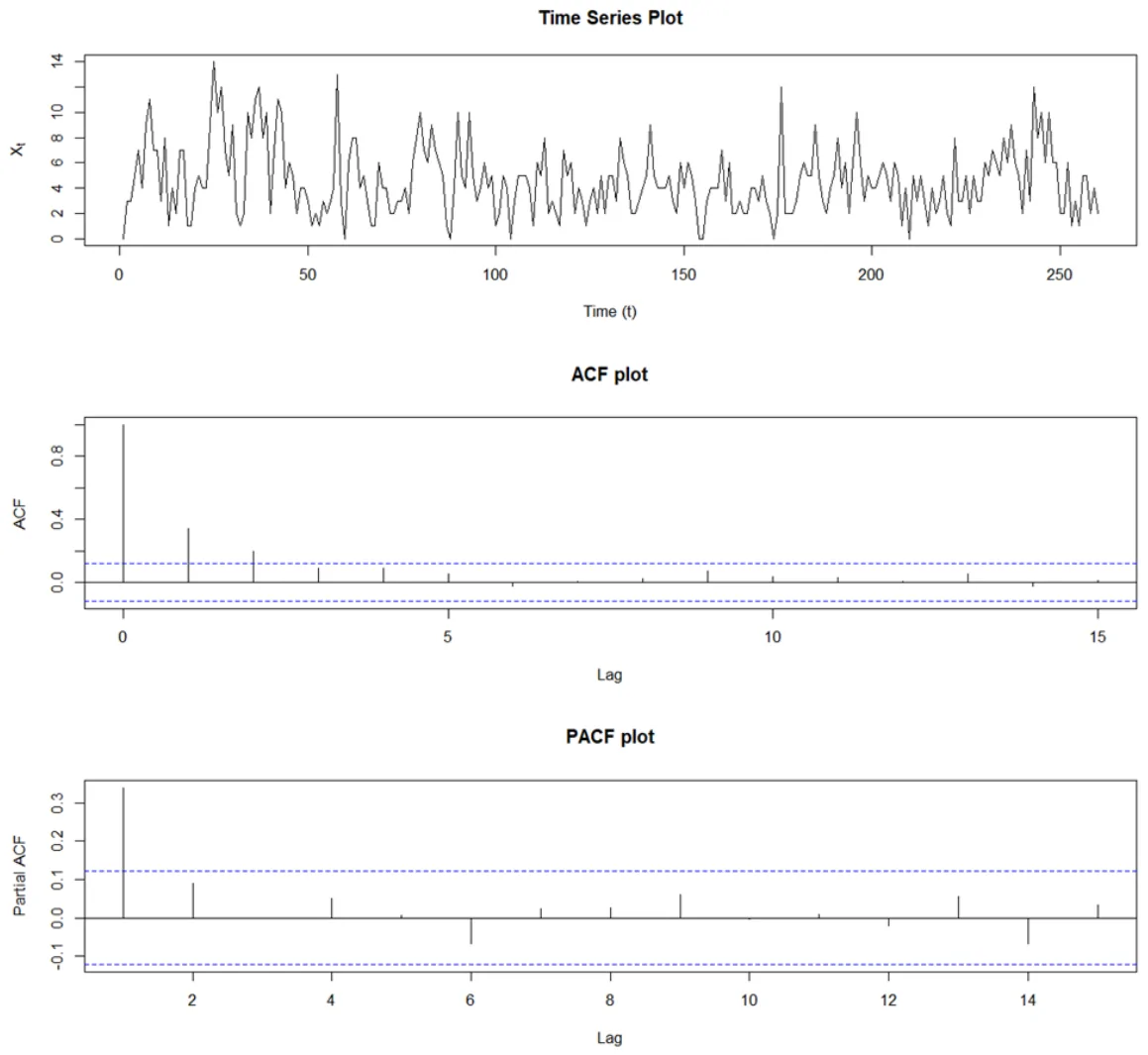
Sample path, sample ACF, and sample PACF plots for *E. coli* enteritis cases.

Figure 10 displays the frequency distribution of the observed counts. The sample mean and variance are 4.5846 and 7.6029, respectively, indicating the presence of overdispersion in the data. The time series plot, together with the sample ACF and PACF shown in Figure 11, does not reveal any noticeable seasonal pattern, suggesting that the series exhibits non-seasonal behavior.

The Euclidean distances between the actual and estimated probabilities for counts zero, one, and two under the geometric, Poisson, transmuted geometric (TGD), zero-modified geometric (ZMG), and zero–one–two modified Poisson (ZOTMPo) marginal distributions are reported in Table 7. Among the considered distributions, the ZOTMPo model gives the smallest Euclidean distance, indicating the best approximation to the observed probabilities.

Parameter estimates and model comparison criteria, including AIC, BIC, and RMSE, for the competing INAR(1) models are presented in Table 8. For the fitted ZOINBINAR(1) process, the estimated zero and one inflation parameters are $\left(\hat{\alpha }=7\times 10^{-7}\right)$ and $\left(\hat{\beta }=1\times 10^{-7}\right)$, respectively. After rounding to four decimal places, both estimates are reported as (0.0000), indicating that the zero and one inflation components contribute negligibly to the fitted ZOINBINAR(1) model. This finding is also consistent with Figure 10, which shows no substantial evidence of excess zeros or ones in the observed data. In contrast, Figure 10 suggests the presence of inflation at the count value two, a phenomenon that cannot be accommodated by the ZOINBINAR(1) model. The proposed ZOTMPo–INAR model explicitly accounts for this excess occurrence of twos and is therefore better suited for modeling the underlying count process.

Although the proposed ZOTMPo–INAR(1) model does not uniformly outperform all competing models on every metric, it shows consistently competitive performance. In particular, it attains the lowest BIC value, indicating a superior balance between goodness-of-fit and model complexity. This is particularly relevant compared to models such as ZOINBINAR(1), which involve a larger number of parameters (five versus four in the proposed model). Thus, the ZOTMPo–INAR(1) model provides a more parsimonious yet effective representation of the data.

**Figure 12:**
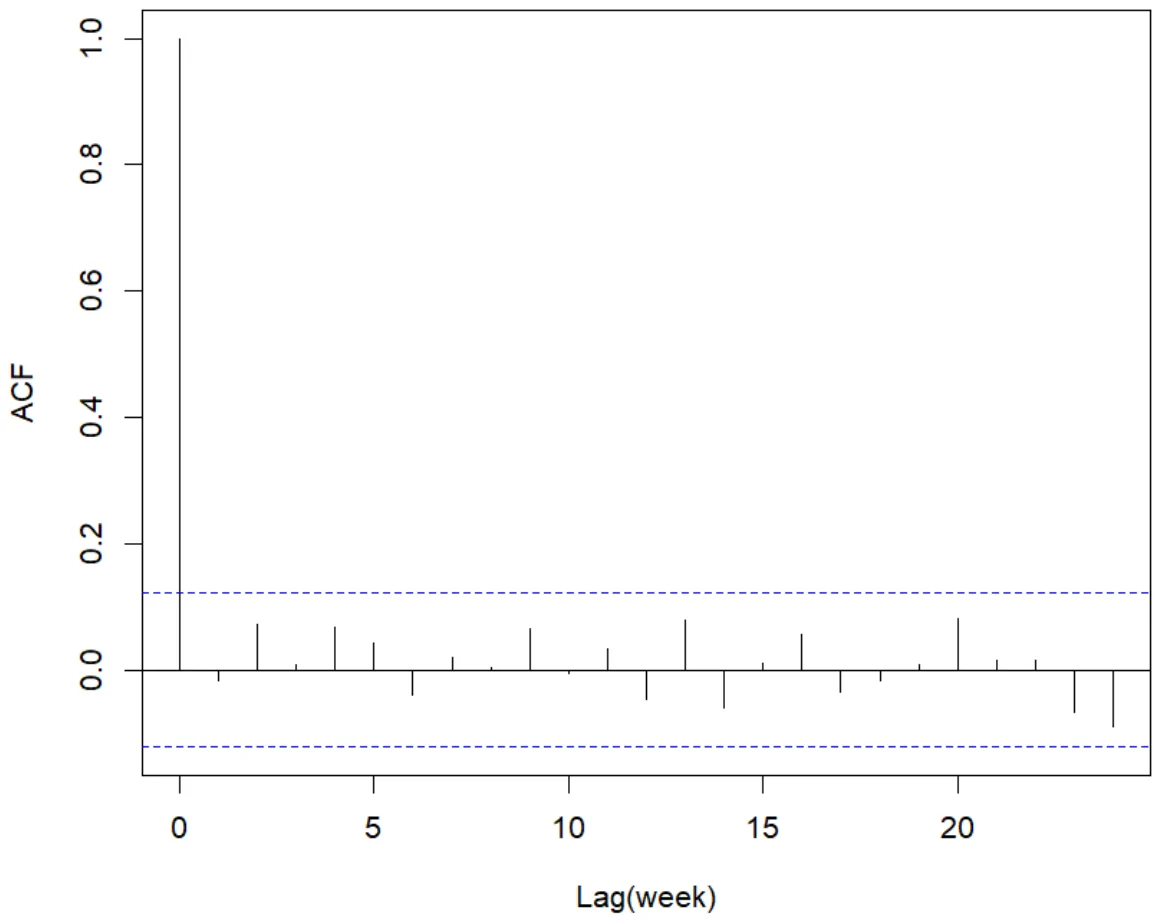
Sample ACF of Pearson residuals for *E. coli* enteritis cases.

**Figure 13:**
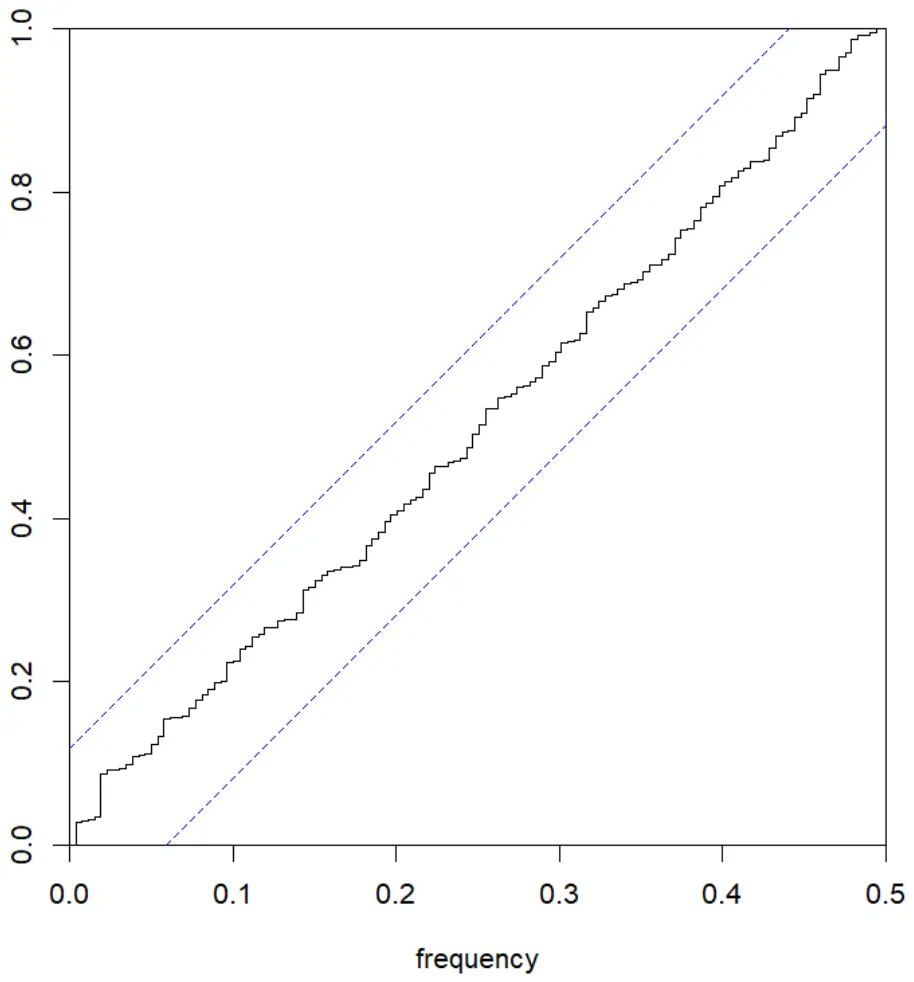
The cumulative periodogram plot of the Pearson residuals for *E. coli* enteritis cases.

The adequacy of the fitted model is further assessed through residual diagnostics. The sample ACF of the Pearson residuals, shown in Figure 12, indicates that there is no significant autocorrelation in the residuals, supporting the suitability of the fitted model. In addition, the cumulative periodogram presented in Figure 13 suggests that the residual series behaves like white noise.

**Figure 14:**
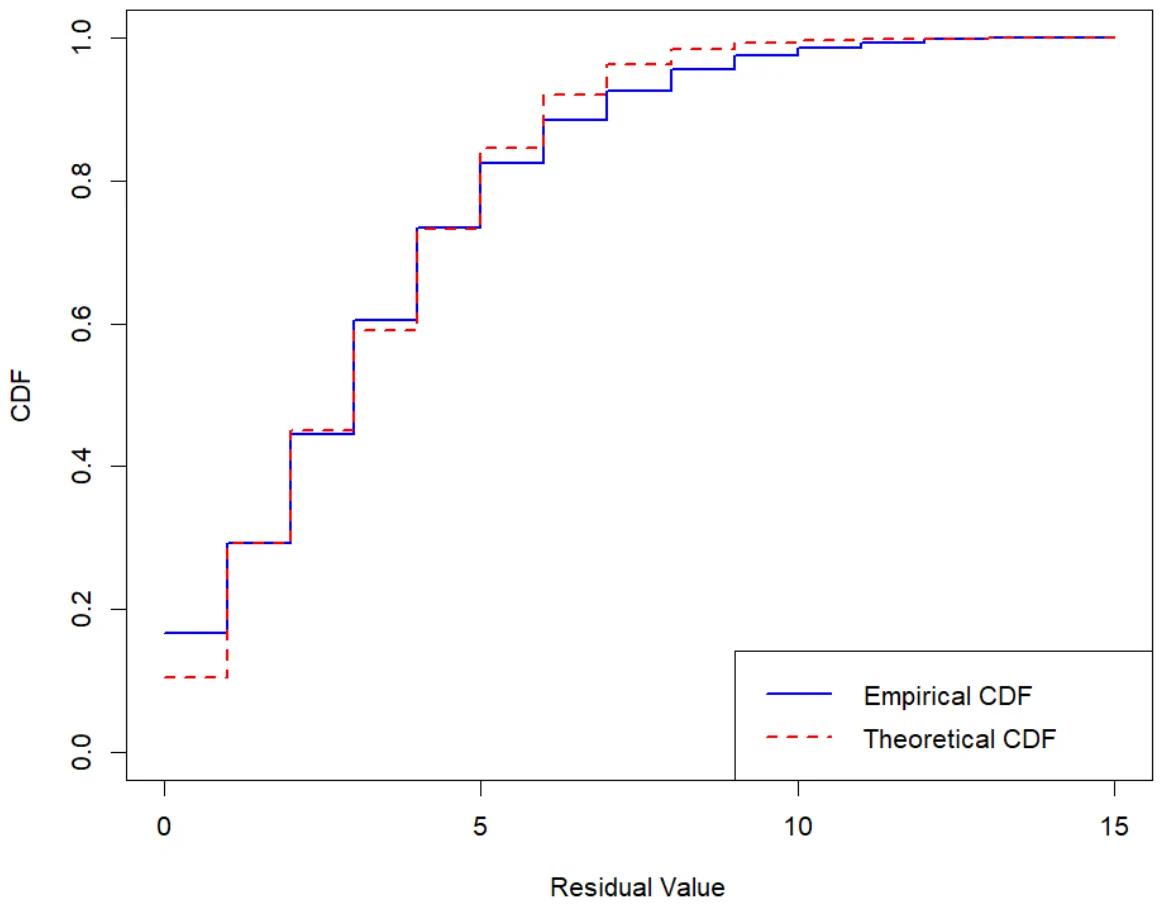
Empirical vs. theoretical CDF of ZOTMPo distribution for *E. coli* cases.

The last ten observations of the dataset were used for forecasting purposes. The resulting multi-step-ahead forecasts from the selected model are reported in Table 9. In addition, Figure 14 presents a comparison between the empirical cumulative distribution function (CDF) of the standardized innovations and the theoretical CDF of the fitted ZOTMPo distribution. The close agreement between these curves indicates that the proposed innovation distribution adequately represents the underlying data-generating mechanism.

**Figure 15:**
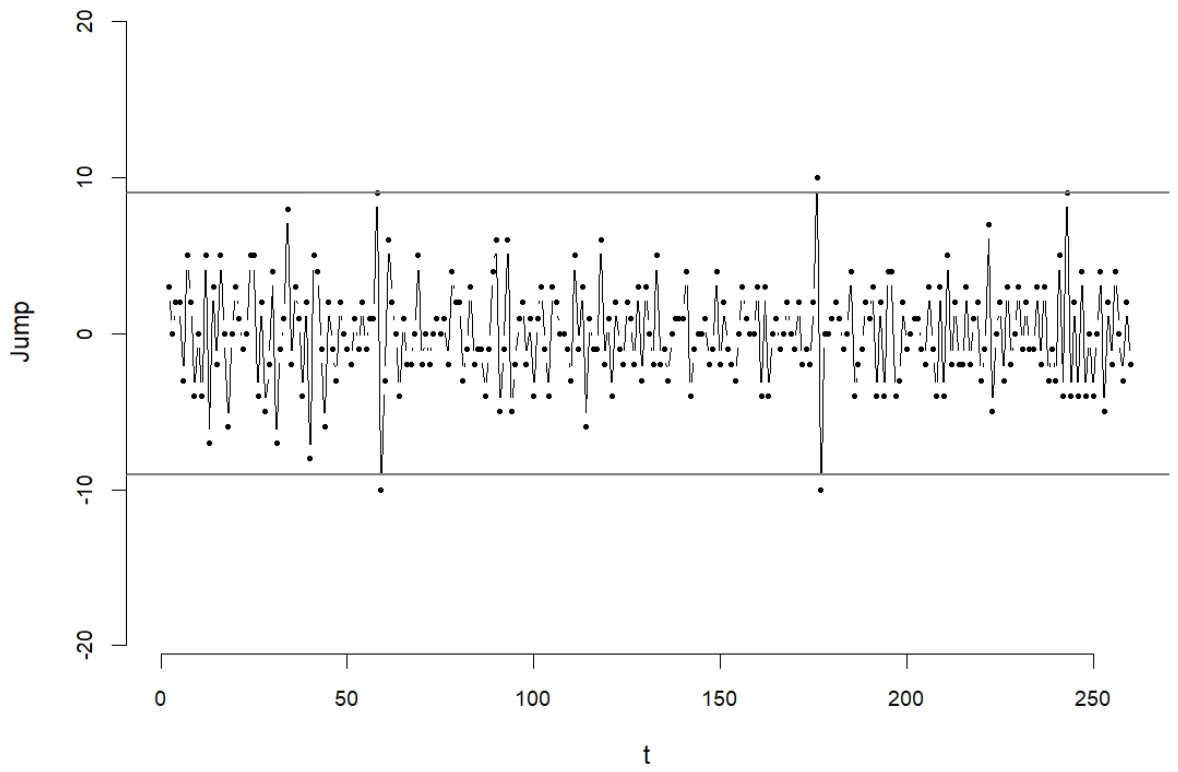
The jumps against time of the proposed model for the *E. coli* enteritis cases.

The jump process for the *E. coli* dataset (Figure 15) fluctuates randomly around zero, most observations lie within the prescribed control limits, and there is no evidence of systematic patterns or clustering. This behavior is consistent with the theoretical properties of a well-specified INAR(1) process and further supports the adequacy of the ZOTMPo–INAR(1) model in capturing the dependence structure and variability in the data.

**Figure 16:**
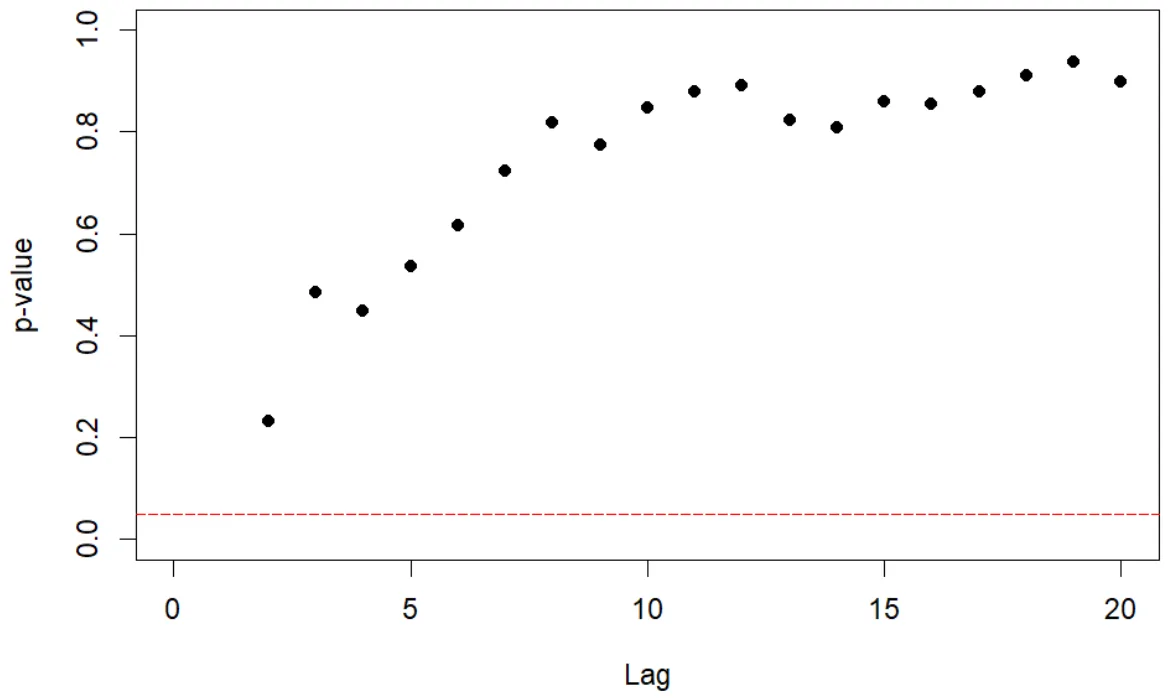
The Ljung–Box *p*–values for *E. coli* enteritis cases.

Moreover, the results of the Ljung–Box test for the residuals (Figure 16) show that the *p*-values remain consistently above the significance level of 0.05 in all the lags considered. This indicates that there is no significant residual autocorrelation, suggesting that the residuals behave approximately as white noise and that the model sufficiently captures the serial dependence in the *E. coli* time series.

## 7. Concluding Remarks

This study proposed a novel first-order integer-valued autoregressive model, the ZOTMPo–INAR(1) process, to address the challenges of modeling overdispersed count time series exhibiting inflation or deflation at low-count values. Unlike existing Poisson-based or single-value modified models, the proposed framework simultaneously adjusts the probabilities of the counts zero, one, and two. This multipoint modification allows the model to capture complex, irregular structural behaviors at these specific states while preserving the standard first-order dependence structure of the INAR(1) process.

The theoretical and finite sample properties of the model were thoroughly investigated. A Monte Carlo simulation study confirmed that the conditional maximum likelihood (CML) estimators perform satisfactorily, exhibiting approximate unbiasedness and decreasing mean squared errors as the sample size increases. Furthermore, we established the information-theoretic properties of the proposed process. By deriving bounds for the Shannon entropy and conditional entropy, the study provides a mathematically rigorous approach to quantifying the dynamic complexity, uncertainty, and predictability of the stochastic system.

The practical utility of the ZOTMPo–INAR(1) model was demonstrated through the analysis of two real-world epidemiological datasets: *E. coli* enteritis and dengue fever cases in Germany. Comprehensive performance assessments utilizing information criteria (AIC, BIC), forecast error measures (RMSE, MAE), probabilistic scoring functions, and the Euclidean distance for low-count probabilities revealed that the proposed model consistently outperformed competing INAR-type models in both model fit and forecasting accuracy. Additionally, rigorous diagnostic checks, including the analysis of Pearson residual autocorrelation functions, cumulative periodograms, Ljung–Box tests, and jump processes, confirmed that the fitted model adequately represents both the marginal distributions and the underlying serial dependence present in the observed data.

Despite its significant advantages, the current study is subject to certain limitations that present opportunities for future research. The proposed model is confined to first-order autoregression and modifies probabilities exclusively at the counts of zero, one, and two, making it potentially less suitable for time series characterized by anomalies at higher count values or more complex temporal dependencies. Future work will focus on extending the proposed framework to higher-order and seasonal INAR models, adapting it for multivariate count time series, and developing new innovation distributions to accommodate inflation or deflation at additional count states.

## Figures and Tables

**Table 1:** Inflation and deflation equations with different orders.

Case	Inflation Equation	Deflation Equation
Zero	ln(1−p)>−λ/2	ln(1−p)<−λ/2
One	(lnλ−λ)<ln(2p(1−p))	(lnλ−λ)>ln(2p(1−p))
Two	$p>\frac{\lambda \sqrt{e^{-\lambda }}}{\sqrt{2}}$	$p<\frac{\lambda \sqrt{e^{-\lambda }}}{\sqrt{2}}$

**Table 2:** Comparison of theoretical (Theo.) and empirical (Emp.) ERL.

	${\mathrm{ERL}}_{0}$		${\mathrm{ERL}}_{1}$		${\mathrm{ERL}}_{2}$	
Parameters	Theo.	Emp.	Theo.	Emp.	Theo.	Emp.
α=0.2, ϕ=0.5, λ=2, p=0.2	1.6331	1.6291	1.4573	1.4589	1.2649	1.2646
α=0.2, ϕ=0.5, λ=2, p=0.5	1.2386	1.2383	1.5309	1.5338	1.4237	1.4201
α=0.2, ϕ=0.5, λ=2, p=0.8	1.0961	1.0954	1.3401	1.3397	1.6378	1.6406

**Table 3:** Parameter estimates and their mean squared errors (in parentheses) using CML.

*n*	$\hat{\alpha }$	$\hat{\phi }$	$\hat{\lambda }$	$\hat{p}$
α=0.2,ϕ=0.7,λ=4,p=0.3				
50	0.1825	0.6737	3.9573	0.2989
	(0.0116)	(0.0124)	(0.6553)	(0.0121)
100	0.1909	0.6923	4.0046	0.2996
	(0.0056)	(0.0055)	(0.3265)	(0.0051)
500	0.1987	0.6974	3.9902	0.2992
	(0.0010)	(0.0010)	(0.0614)	(0.0010)
1000	0.1992	0.6994	3.9950	0.2995
	(0.0005)	(0.0005)	(0.0282)	(0.0005)
α=0.8,ϕ=0.5,λ=3,p=0.7				
50	0.7948	0.4735	3.1458	0.7390
	(0.0025)	(0.0577)	(0.7752)	(0.0909)
100	0.7956	0.4604	3.0958	0.7353
	(0.0014)	(0.0487)	(0.5004)	(0.0774)
500	0.7977	0.4713	2.9944	0.7162
	(0.0005)	(0.0176)	(0.1293)	(0.0294)
1000	0.7991	0.4866	2.9938	0.7075
	(0.0003)	(0.0088)	(0.0705)	(0.0147)
α=0.2,ϕ=0.1,λ=7,p=0.1				
50	0.1649	0.1086	7.3140	0.2715
	(0.0130)	(0.0037)	(0.8250)	(0.1438)
100	0.1760	0.1054	7.1864	0.2094
	(0.0070)	(0.0018)	(0.4335)	(0.0751)
500	0.1951	0.1009	7.0450	0.1174
	(0.0011)	(0.0003)	(0.0731)	(0.0161)
1000	0.1979	0.0993	7.0181	0.1087
	(0.0006)	(0.0001)	(0.0403)	(0.0083)

**Table 4:** Euclidean distance between actual and estimated probabilities (dengue data).

Distribution	Geometric	Poisson	TGD	ZMG	ZOTMPo
Euclidean distance	0.2056	0.0685	0.0788	0.1119	0.0285

**Table 5:** Model comparison based on information criteria and forecast accuracy measures for dengue fever data.

Model	Estimates	AIC	BIC	RMSE	MAE	SLS	SQS	SRPS
PoINAR(1)	$\hat{\phi }=0.1589,\hat{\lambda }=2.9904$	728.2307	734.4666	2.2788	1.8090	2.1814	−0.1307	1.2522
NGINAR(1)	$\hat{\alpha }=0.5816,\hat{\mu }=3.0975$	767.9327	774.1687	2.4235	1.9298	2.3010	−0.1005	1.3836
GINAR(1)	$\hat{\phi }=0.3867,\hat{\theta }=0.7375$	772.9710	779.2070	2.3511	1.8190	2.3162	−0.0952	1.3766
ZMGINAR(1)	$\hat{\alpha }=0.1940,\hat{\pi }=-0.3647,$ $\hat{\mu }=2.4787$	731.8640	741.2180	2.2792	1.7883	2.1863	−0.1253	1.2703
TGDINAR(1)	$\hat{\alpha }=-0.9999,\hat{q}=0.6852,$ $\hat{\lambda }=0.2545$	731.4102	740.7642	2.2693	1.8029	2.1850	−0.1326	1.2600
ZOINBINAR(1)	$\hat{\rho }=0.2232,\hat{\alpha }=0.0000,$ $\hat{\beta }=0.1562,\hat{r}=3.1735,$ $\hat{p}=0.49999$	720.0058	735.5958	2.2683	1.8214	2.1386	−0.1391	1.2406
ZOTMPo–INAR(1)	$\hat{\alpha }=0.1928,\hat{\phi }=0.3952,$ $\hat{\lambda }=3.9545,\hat{p}=0.6050$	715.5899	728.0619	2.2682	1.8072	2.1313	−0.1393	1.2364

**Table 6:** Multi-step-ahead forecasts for the dengue fever dataset.

Forecast Horizon (*i*)	Actual Observed Value	Mean Forecast	Median Forecast	Mode Forecast
1	2	3.06	3	2
2	3	3.45	3	2
3	0	3.53	3	2
4	5	3.54	3	2
5	8	3.55	3	2
6	3	3.55	3	2
7	3	3.55	3	2
8	9	3.55	3	2
9	2	3.55	3	2
10	5	3.55	3	2

**Table 7:** Euclidean distance between actual and estimated probabilities (*E. coli* enteritis).

Distribution	Geometric	Poisson	TGD	ZMG	ZOTMPo
Euclidean distance	0.1693	0.0494	0.0840	0.1524	0.0283

**Table 8:** Model comparison based on information criteria and forecast accuracy measures for *E. coli* data.

Model	Estimates	AIC	BIC	RMSE	MAE	SLS	SQS	SRPS
PoINAR(1)	$\hat{\phi }=0.2518,\hat{\lambda }=3.4456$	1224.7770	1231.8984	2.5889	1.9939	2.3567	−0.1143	1.4345
NGINAR(1)	$\hat{\alpha }=0.7230,\hat{\mu }=4.1603$	1266.9184	1274.0397	2.7817	2.1786	2.4381	−0.0897	1.5708
GINAR(1)	$\hat{\phi }=0.4744,\hat{\theta }=0.7656$	1312.7740	1319.8954	2.6977	2.0402	2.5266	−0.0637	1.6141
ZMGINAR(1)	$\hat{\alpha }=0.5430,\hat{\pi }=-0.2139,$ $\hat{\mu }=3.3221$	1258.1987	1268.8807	2.6502	2.0236	2.4174	−0.0893	1.5110
TGDINAR(1)	$\hat{\alpha }=-1.0000,\hat{q}=0.7333,$ $\hat{\lambda }=0.3830$	1237.8837	1248.5657	2.5805	1.9722	2.3782	−0.1026	1.4647
ZOINBINAR(1)	$\hat{\rho }=0.3251,\hat{\alpha }=0.0000,$ $\hat{\beta }=0.0000,\hat{r}=3.1419,$ $\hat{p}=0.4999$	1203.4878	1221.2912	2.5730	1.9870	2.3040	−0.1173	1.4152
ZOTMPo–INAR(1)	$\hat{\alpha }=0.3216,\hat{\phi }=0.2764,$ $\hat{\lambda }=3.9902,\hat{p}=0.4296$	1206.6932	1220.9359	2.5729	1.9820	2.3141	−0.1158	1.4173

**Table 9:** Multi-step-ahead forecasts for *E. coli* enteritis cases.

Forecast Horizon (*i*)	Actual Observed Value	Mean Forecast	Median Forecast	Mode Forecast
1	2	3.77	4	2
2	6	4.33	4	3
3	1	4.51	4	4
4	3	4.57	4	4
5	1	4.59	4	4
6	5	4.60	4	4
7	5	4.60	4	4
8	2	4.60	4	4
9	4	4.60	4	4
10	2	4.60	4	4

## Data Availability

Datasets analyzed in this study can be directly accessed via the web links cited within the respective data analysis section of this paper.

## Appendix A. Proof of Theorem 1

**Proof.** By recursively expanding Equation (3) and utilizing the property of the binomial thinning operator, for *k* $\in N_{0}$, we can write

(A1) $$X_{t}=\alpha^{k}*X_{t-k}+\sum_{j=0}^{k-1}\alpha^{j}*\varepsilon_{t-j}\;.$$

Using the above equation, we adjust the terms and take their expectation after squaring them on both sides,

$$\begin{array}{c} E{\left[X_{t}-\sum_{j=0}^{k-1}\alpha^{j}*\varepsilon_{t-j}\right]}^{2}=E{\left[\alpha^{k}*X_{t-k}\right]}^{2}. \end{array}$$

Conditioning on $X_{t}$, the distribution of the entity $\alpha *X_{t}|X_{t}$ becomes Bin$\left(X_{t},\alpha \right)$, and the distribution of $\alpha^{k}*X_{t}|X_{t}$ is Bin$\left(X_{t},\alpha^{k}\right)$. The pgf of the random variable $\alpha^{k}*X_{t}|X_{t}$ is given as follows:

(A2) $$\Phi_{\alpha^{k}*X_{t}|X_{t}}\left(s\right)={\left(1-\alpha^{k}+\alpha^{k}s\right)}^{X_{t}}.$$

The pgf of the random variable $\alpha^{2}*X_{t}|X_{t}$ is given as follows:

$$\begin{array}{c} \Phi_{\alpha *\alpha *X_{t}|X_{t}}\left(s\right)={\left(1-\alpha^{2}+\alpha^{2}s\right)}^{X_{t}}. \end{array}$$

Using the properties of the PGF (evaluating its derivatives at *s* = 1), the moments can be derived as follows:

$$\begin{array}{c} E\left(\alpha^{2}*X_{t}|X_{t}\right)=\alpha^{2}X_{t}\;\;\;\mathrm{and}\;\;\;Var\left(\alpha^{2}*X_{t}|X_{t}\right)=\alpha^{2}\left(1-\alpha^{2}\right)X_{t}\;. \end{array}$$

The pgf of the random variable $\alpha^{3}*X_{t}|X_{t}$ is given as follows:

(A3) $$\begin{array}{c} \Phi_{\alpha *\alpha *\alpha *X_{t}|X_{t}}\left(s\right)={\left(1-\alpha^{3}+\alpha^{3}s\right)}^{X_{t}}. \end{array}$$

The moments are as follows:

$$\begin{array}{c} E\left(\alpha^{3}*X_{t}|X_{t}\right)=\alpha^{3}X_{t}\;\;\;\mathrm{and}\;\;\;Var\left(\alpha^{3}*X_{t}|X_{t}\right)=\alpha^{3}\left(1-\alpha^{3}\right)X_{t}\;\;. \end{array}$$

Using the pgf, moments can be easily derived, and we can find the second moment from the (A3) as follows:

$$\begin{array}{cc} E{\left[(\alpha^{k}*X_{t}\right)}^{2}]=E\left[E{\left(\alpha^{k}*X_{t}\right)}^{2}|X_{t}\right]\; & =E\left[\mathrm{Var}\left(\alpha^{k}*X_{t}|X_{t}\right)+{\left(E(\alpha^{k}*X_{t}|X_{t})\right)}^{2}\right]\\ & =E\left[\alpha^{k}\left(1-\alpha^{k}\right)X_{t}+\alpha^{2k}X_{t}^{2}\right]\\ & =\alpha^{k}\left(1-\alpha^{k}\right)E\left(X_{t}\right)+\alpha^{2k}E\left(X_{t}^{2}\right)\\ & =\alpha^{k}\left(1-\alpha^{k}\right)\mu_{X}+\alpha^{2k}\left(\sigma_{X}^{2}+\mu_{X}^{2}\right)\\ & \to 0\;\mathrm{as}\;k\to \infty \end{array}$$

where $\mu_{X}$ and $\sigma_{X}^{2}$ are the mean and variance of the stationary distribution. This proves that the process $\left\{X_{t}\right\}$ converges to $\sum_{j=0}^{\infty }\alpha^{j}*\varepsilon_{t-j}$ in distribution. □
